# TMED inhibition suppresses cell surface PD-1 expression and overcomes T cell dysfunction

**DOI:** 10.1136/jitc-2024-010145

**Published:** 2024-11-07

**Authors:** David W Vredevoogd, Georgi Apriamashvili, Pierre L Levy, Sanju Sinha, Zowi R Huinen, Nils L Visser, Beaunelle de Bruijn, Julia Boshuizen, Susan E van Hal-van Veen, Maarten A Ligtenberg, Onno B Bleijerveld, Chun-Pu Lin, Judit Díaz-Gómez, Santiago Duro Sánchez, Ettai Markovits, Juan Simon Nieto, Alex van Vliet, Oscar Krijgsman, Gal Markel, Michal J Besser, Maarten Altelaar, Eytan Ruppin, Daniel S Peeper

**Affiliations:** 1 Department of Molecular oncology and immunology, Netherlands Cancer Institute, Oncode Institute, Amsterdam, The Netherlands; 2 Cancer Data Science Laboratory, National Cancer Institute Center for Cancer Research, Bethesda, Maryland, USA; 3 Ella Lemelbaum Institute for Immuno-oncology, Sheba Medical Center, Tel Hashomer, Israel; 4 Department of Clinical Microbiology and Immunology, Faculty of Medical & Health Sciences, Tel Aviv University, Tel Aviv, Israel; 5 Davidoff Center and Samueli Integrative Cancer Pioneering Center, Rabin Medical Center, Petah Tikva, Israel; 6 Felsenstein Medical Research Center, The Sackler School of Medicine, Tel Aviv University, Tel Aviv, Israel; 7 Biomolecular Mass Spectrometry and Proteomics, Bijvoet Center for Biomolecular Research and Utrecht Institute for Pharmaceutical Sciences, Utrecht University, Utrecht, The Netherlands

**Keywords:** Immune checkpoint inhibitor, Immunotherapy, T cell, Adoptive cell therapy - ACT, Tumor infiltrating lymphocyte - TIL

## Abstract

**Background:**

Blockade of the programmed cell death protein 1 (PD-1) immune checkpoint (ICB) is revolutionizing cancer therapy, but little is known about the mechanisms governing its expression on CD8 T cells. Because PD-1 is induced during activation of T cells, we set out to uncover regulators whose inhibition suppresses PD-1 abundance without adversely impacting on T cell activation.

**Methods:**

To identify PD-1 regulators in an unbiased fashion, we performed a whole-genome, fluorescence-activated cell sorting (FACS)-based CRISPR-Cas9 screen in primary murine CD8 T cells. A dual-readout design using the activation marker CD137 allowed us to uncouple genes involved in PD-1 regulation from those governing general T cell activation.

**Results:**

We found that the inactivation of one of several members of the TMED/EMP24/GP25L/p24 family of transport proteins, most prominently TMED10, reduced PD-1 cell surface abundance, thereby augmenting T cell activity. Another client protein was cytotoxic T lymphocyte-associated protein 4 (CTLA-4), which was also suppressed by TMED inactivation. Treatment with TMED inhibitor AGN192403 led to lysosomal degradation of the TMED-PD-1 complex and reduced PD-1 abundance in tumor-infiltrating CD8 T cells (TIL) in mice, thus reversing T cell dysfunction. Clinically corroborating these findings, single-cell RNA analyses revealed a positive correlation between TMED expression in CD8 TIL, and both a T cell dysfunction signature and lack of ICB response. Similarly, patients receiving a TIL product with high TMED expression had a shorter overall survival.

**Conclusion:**

Our results uncover a novel mechanism of PD-1 regulation, and identify a pharmacologically tractable target whose inhibition suppresses PD-1 abundance and T cell dysfunction.

**WHAT IS ALREADY KNOWN ON THIS TOPIC**

- Programmed cell death protein-1 (PD-1) is expressed on activated T cells, governed by transcription factors including NFATc1 and AP-1, which also drive other T cell functions such as cytokine production. Recognizing the link between PD-1 expression and T cell activation, it is unclear whether pharmacologically tractable PD-1 regulators exist whose inhibition does not adversely affect general T cell activation.

**WHAT THIS STUDY ADDS**

- We conducted a dual marker fluorescence-activated cell sorting (FACS)-based genomic CRISPR-Cas9 screen to identify specific regulators of PD-1 abundance on primary CD8 T cells, revealing several TMED family chaperones as top hits. Genetic or pharmacologic inactivation of TMED proteins reversed dysfunction of T cells in culture and in mice. TMED inhibition reduced PD-1 protein levels without impeding general T cell activation.

**HOW THIS STUDY MIGHT AFFECT RESEARCH, PRACTICE OR POLICY**

- The discovery that the TMED family regulates PD-1 cell surface abundance, coupled with its susceptibility to small molecule intervention, provides fundamental insight into the mechanisms controlling a crucial inhibitory receptor in immunotherapy while presenting a potential translational opportunity.

## Introduction

 Immune checkpoint blockade (ICB) therapy has proven to be a transformative treatment option for a variety of cancers.^1^,^5^ By blocking inhibitory receptors, most commonly programmed cell death protein-1 (PD-1), programmed death-ligand 1 (PD-L1) or cytotoxic T-lymphocyte associated protein 4 (CTLA-4), T cell function is improved, thereby enabling these cells to more effectively combat a patient’s tumor.^6^,^9^ Even though the clinical benefit of ICB is unprecedented, the majority of patients fail to respond durably to this treatment, because of several types of resistance.^5 10 11^

Lack of ICB efficacy correlates with the accumulation of a functionally altered repertoire of CD8 T cells in the tumor.^12^,^16^ These dysfunctional, “exhausted” T cells, so termed for their limited cytotoxic activity, are characterized by their high expression of inhibitory receptors, including PD-1, LAG3, TIM3 and CD39.^1217^,^23^ While early T cell dysfunction seems to be reversible,^24^,^26^ through concerted activities of transcriptional regulators TCF1, TOX and the NR4A protein family, this dysfunctional state is eventually epigenetically enforced and irreversible.^27^,^40^ This enforcement of exhaustion is also, at least in part, due to PD-1, because anti-PD-(L)1 therapy reinvigorates the exhausted T cell pool in both tumor and viral models.^8 14 26^ Conversely, PD-1 also protects early dysfunctional T cells from hyperstimulation-induced cell death, thereby limiting the onset of terminal exhaustion.^29 33 41^

PD-1 thus plays a dichotomous role in determining the cytotoxic potential of CD8 T cells: limiting T cell activity directly by inhibiting T cell receptor (TCR)-driven signaling and enforcing T cell exhaustion on the one hand, yet maintaining a polyfunctional, cytotoxic T cell repertoire on the other. Despite this key role of PD-1 in T cell antitumor activity, our understanding of the regulation of its expression is incomplete.

PD-1 is expressed on the surface of activated T cells, following the establishment of a permissive chromatin state.^7 13 30 42^ From this accessible chromatin, PD-1 transcription is initiated by a number of transcription factors, including NFATc1 and AP-1, which simultaneously drive other T cell effector functions, such as cytokine production.^43^,^47^ PD-1 transcription can also be repressed, for example, by PRDM1, but also those repressors influence T cell activation status more broadly.^48^,^52^ PD-1 is regulated also post-transcriptionally, for example, by fucosylation through FUT8, which contributes to its stability.^53^ Importantly, however, inhibition or inactivation of FUT8 not only stabilizes PD-1, but also attenuates TCR signaling.^54^ These mechanistic studies reinforce the notion that PD-1 expression and T cell activation are inextricably linked. This prompted us to uncover new and specific PD-1-regulatory factors, which do not negatively affect general T cell activation and which may be pharmacologically tractable. We therefore conducted a genome-wide genetic screen in primary T cells aiming to find factors that alter PD-1 abundance without negatively affecting T cell activation.

## Results

### Whole-genome dual marker fluorescence-activated cell sorting-based CRISPR-Cas9 screen in primary murine CD8 T cells identifies TMED proteins as critical regulators of PD-1 protein abundance

To identify specific PD-1 regulators, we performed a dual-marker, whole-genome, fluorescence-activated cell sorting (FACS)-based CRISPR-Cas9 knockout (KO) screen in primary murine CD8 T cells. We first crossed mice constitutively expressing Cas9-EGFP^55^ to mice genetically engineered to express a TCR specific to ovalbumin_257–264_ (OVA) in the context of H2-Kb (OT-I TCR).^56^ From these newly established OT-I/Cas9 mice we isolated CD8 T cells with a defined specificity for OVA (online supplemental figure 1a). We confirmed by flow cytometry that activation of these cells with an agonistic CD3 antibody led to the upregulation of both PD-1 and the activation marker CD137. KO of *Pdcd1* (the gene encoding PD-1) resulted in reduced PD-1 abundance, serving as a validation for successful CRISPR-Cas9 perturbation of these primary cells (online supplemental figure 1b–e and online supplemental table 5).^57^ In parallel, we generated a retroviral, whole-genome CRISPR-Cas9 sgRNA library by subcloning the Brie library^58^ into a retroviral backbone. We confirmed by deep sequencing that this library had maintained high complexity and uniformity (online supplemental figure 1f and online supplemental table 1).

**Figure 1:**
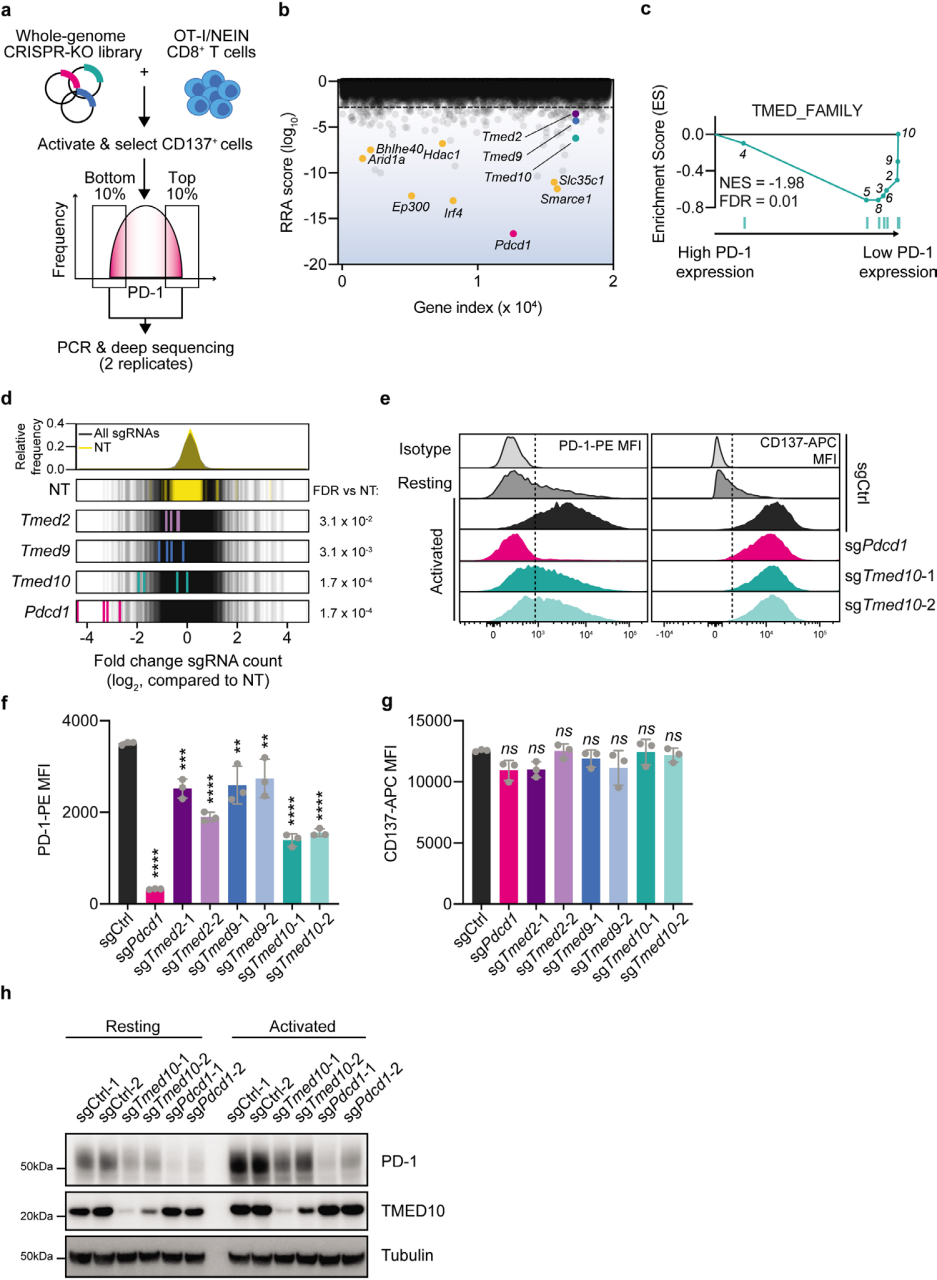
Whole-genome dual marker FACS-based CRISPR-Cas9 screen in primary murine CD8 T cells identifies TMED proteins as regulators of PD-1 abundance. (**a**) Schematic representation of the dual-marker FACS-based CRISPR-Cas9 screen for PD-1 regulators. (**b**) RRA score (log_10_) for each of the genes in the PD-1 regulator screen comparing the PD-1^High^ to the PD-1^Low^ populations. The gene index value refers to the numerical position of a given gene in an alphabetized list of all targeted genes. Statistics were performed by MAGeCK (V0.5.7).^63^ (**c**) Gene set enrichment analysis plot of the TMED_FAMILY gene set, comprising all known TMED protein family members, using the RRA score from (**b**) as a ranking metric. The numbers refer to the position of each individual TMED family member. (**d**) Log_2_-fold change of individual sgRNAs compared with all sgRNAs. Each of the guides targeting a particular gene is highlighted, with its values being normalized to the non-targeting sgRNA distribution. Statistics were performed by MAGeCK (V.0.5.7).^63^ The dotted red line indicates a twofold change. (**e**) Representative flow cytometry histograms for the abundance of PD-1-PE (left) and CD137-APC in OT-I/Cas9 CD8 T cells of the indicated genotypes and activation status. The dotted line indicates the level of the background signal. (**f**) Quantification of the experiment in (**d**) for PD-1-PE MFI of activated cells. Each data point indicates data obtained with CD8 T cells from an independent spleen. Error bars denote SD. Statistics were performed with a one-way analysis of variance, followed by a Dunnett post hoc test, comparing the MFI of each of the populations to that of the non-targeting control sgRNA condition. (**g**) As in (**e**) but for CD137-APC MFI. (**h**) Western blot analysis of PD-1 and TMED10 abundance in OT-I/Cas9 CD8 T cells carrying the indicated sgRNA before or after activation with CD3 antibody for 24 hours. The size markings indicate the size of the closest molecular weight marker. *p<0.05; **p<0.01; ***p<0.001; ****p<0.0001. FACS, fluorescence activated cell sorting; MFI, mean fluorescence intensity; PD-1, programmed cell death protein-1.

To perform the screen, we activated and transduced CD8 T cells with the library in two replicates. After 2 days of puromycin selection to enrich for successfully transduced cells, we harvested a library reference sample to be used for later analyses. After a further 7 days, we harvested a bulk sample, and activated the remaining cells with CD3 antibody. We considered the possibility that this screen could also pick up genes whose inactivation prevents T cell activation altogether. To prevent this, and to identify specific PD-1 regulators that are not required for general T cell activation, we performed FACS to select the subpopulation of cells that were positive for CD137 after 24 hours. This marker (also named 4-1BB) is a member of the tumor necrosis factor receptor (TNFR) family with a co-stimulatory function that is induced in activated T cells.^59^,^61^ We then sorted the 10% of cells expressing the highest and lowest levels of PD-1, respectively. This dual-marker screen design allowed us to disentangle proteins involved in PD-1 regulation from those involved in more general T cell activation. Lastly, we isolated the genomic DNA from the harvested cell populations and identified the sgRNAs they harbored by deep sequencing (figure 1a and online supplemental table 1).

To assess the quality of the screen, we first compared the sgRNA distribution in the two replicates of each sample. We found a strong correlation between replicates in each of the screen arms (online supplemental figure 1g). To confirm successful gene targeting by Cas9, we analyzed the relative depletion of both non-essential and essential genes^62^ in the library reference and bulk samples by MAGeCK analysis.^63^ We found a strong depletion of core essential genes, confirming successful Cas9 targeting and highlighting the quality of the screen (online supplemental figure 1h–j and online supplemental table 1). Next, we compared the PD-1^High^ and PD-1^Low^ populations, again by MAGeCK analysis. As the top hit decreasing abundance of PD-1, we identified sgRNAs targeting *Pdcd1* itself. Aside from this expected hit, we also identified a number of previously characterized PD-1 regulators, including *Irf4* (encoding a major driver of T cell exhaustion),^38^ *Ep300,*^64^ *Smarce1,*^65 66^ *Slc35c1,*^53^ *Arid1a*^65 66^*, Bhlhe40*^67^ and *Hdac1*.^43^ As potential new PD-1 regulators, we identified multiple members of the TMED/EMP24/GP25L/p24 protein family, specifically *Tmed2, Tmed9 and Tmed10*, which have not yet been described in this context, with the family being enriched among hits (figure 1b–d and online supplemental table 1). The TMED proteins, often found in hetero-oligomeric complexes, are involved in the vesicular trafficking of specific proteins between the endoplasmic reticulum and the Golgi apparatus.^68^ The fact that multiple members of the same protein family are identified as hits made us particularly interested in their validation, characterization and clinical relevance.

We validated these hits in an arrayed fashion: we transduced OT-I/Cas9 CD8 T cells with single sgRNAs targeting different members of the TMED protein family, or a non-targeting control. We then activated cells with CD3 antibody overnight and assessed their abundance of PD-1 and CD137 by flow cytometry. This analysis confirmed that the deletion with either of two independent sgRNAs of each of the *Tmed* genes led to a substantially reduced abundance of PD-1, without affecting CD137 abundance (figure 1e–g, online supplemental figure 1k,l, 9 and online supplemental table 5). A particularly strong effect was noted for the KO of *Tmed10*, which resulted in a twofold reduction in PD-1 abundance.

To rule out that the reduced detection of PD-1 by flow cytometry was due to a technical artifact, such as reduced antibody binding by differential glycosylation,^69^ we labeled the cells with a fusion protein comprising the extracellular domain of PD-L1 and a human antibody Fc domain (PD-L1-Fc). This enabled us to quantify the binding of PD-L1 to the T cells by staining with a fluorescent secondary antibody targeting the human Fc domain. Performing this experiment showed us that *Tmed10* KO cells displayed reduced binding of PD-L1 when compared with wild-type (WT) cells, corroborating the flow cytometry data (online supplemental figure 1m). In line with this, by analyzing *Tmed10* KO cells by western blot, we found not only reduced abundance of PD-1, but also confirmed successful functional perturbation of *Tmed10* and *Pdcd1* by Cas9 (figure 1h).

The depletion experiments show that cell surface abundance of PD-1 depends on the presence of TMED10. To examine whether also the converse is true, we ectopically expressed *Tmed10* in OT-I/Cas9 CD8 T cells. Flow cytometry and western blotting revealed that, indeed, this led to the induction of PD-1 (online supplemental figure 1n,o). These results together demonstrate that the deletion of *Tmed10* reduces PD-1 protein abundance in CD8 T cells without negatively affecting their activation.

### *Tmed10* deletion enhances CD8 T cell activation by impairing PD-1 trafficking to the cell surface

Next, we aimed to understand the mechanism by which the identified TMED proteins regulate PD-1 abundance, first assessing whether their deletion alters the messenger RNA (mRNA) levels of the latter. We activated either WT or *Tmed10* KO cells with CD3 antibody for 24 hours, and then analyzed the expression of the *Pdcd1* transcript by quantitative PCR (qPCR). This showed that the KO of none of the tested TMED genes significantly altered *Pdcd1* mRNA levels (online supplemental figure 2a), implying that they regulate PD-1 in a post-transcriptional manner.

**Figure 2:**
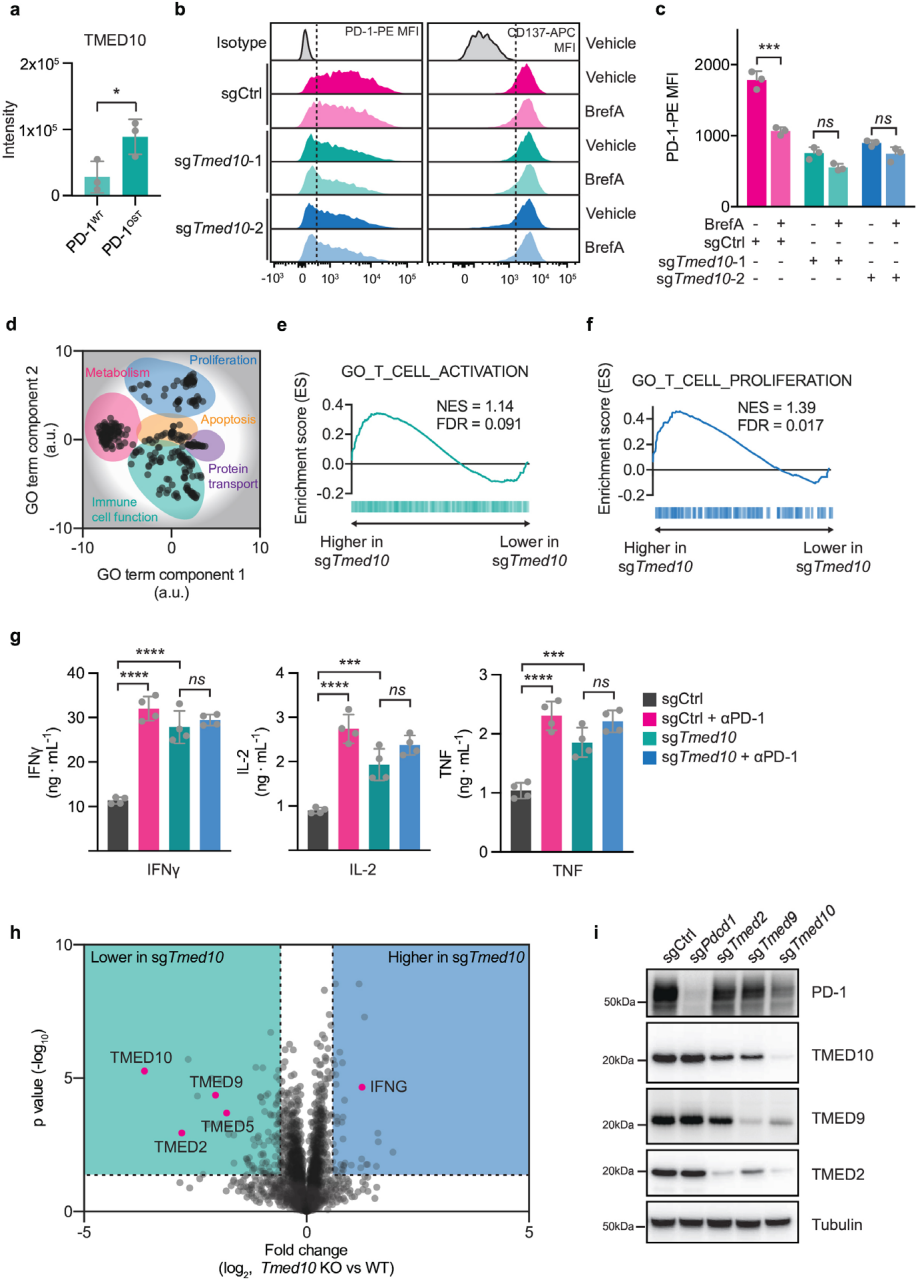
Tmed10 deletion enhances CD8 T cell activation by impairing PD-1 trafficking to the cell surface. (**a**) Quantification of mass spectrometric readout of PD-1^OST^ co-immunoprecipitations^76^ in murine T cells. Each data point indicates the results obtained from a single immunoprecipitation. Error bars denote SD. Statistical analysis was performed with a Student’s t-test. (**b**) Representative flow cytometry histograms for the abundance of PD-1-PE (left) and CD137-APC (right) in OT-I/Cas9 CD8 T cells of the indicated genotypes and treatment. The dotted line indicates the level of the background signal. (**c**) Quantification by flow cytometry of PD-1 abundance on OT-I/Cas9 CD8 T cells carrying the indicated sgRNAs in the presence or absence of brefeldin A (200 ng/mL). Each data point indicates data obtained with CD8 T cells from an independent spleen. Error bars denote SD. Statistical analysis was performed with a one-way ANOVA, followed by a Tukey post hoc test. (**d**) REVIGO^80^ representation of significant gene ontology terms. a.u., arbitrary units. (**e**) Gene Set Enrichment Analysis plot of the gene set GO_T_CELL_ACTIVATION comparing activated OT-I/Cas9 cells carrying an sgRNA targeting *Tmed10*, or a non-targeting control. (**f**) As in (**d**) but for the gene set GO_T_CELL_PROLIFERATION. (**g**) Cytokine release of IFN-γ (left), IL-2 (middle) and TNF (right) as measured by cytometric bead array of sgCtrl or sg*Tmed10* OT-I/Cas9 CD8 T cells, after co-culture with B16F10-OVA tumor cells in a 1:4 ratio in the presence or absence of PD-1 antibody (10 µg/mL). Each data point indicates data obtained with CD8 T cells from an independent spleen. Error bars denote SD. Statistical analysis was performed with a one-way ANOVA, followed by a Tukey post hoc test. (**h**) Proteomic differences between OT-I/Cas9 CD8 T cells carrying either an sgRNA targeting *Tmed10* or a non-targeting control sgRNA after activation with CD3 antibody for 24 hours as measured by mass spectrometry. The data is based on three independent spleens for each genotype. Statistical analysis was performed by a Student’s t-test. (i) Western blot analysis of PD-1, TMED10, TMED9 and TMED2 in OT-I/Cas9 CD8 T cells carrying the indicated sgRNA construct after activation with CD3 antibody for 24 hours. The size markings indicate the size of the closest molecular weight marker. *p<0.05; **p<0.01; ***p<0.001; ****p<0.0001. ANOVA, analysis of variance; FDR, false discovery rate; IFN, interferon; IL, interleukin; NES, normalized enrichment score; PD-1, programmed cell death protein-1; TNF, tumor necrosis factor.

As TMED10 in particular is known to regulate the vesicular trafficking and stability of a number of proteins to and in the cell surface,^6870^,^75^ we next investigated whether it regulates PD-1 in this manner. We first wanted to confirm that TMED10 is associated with proteins in the plasma membrane of CD8 T cells. We labeled intact CD8 T cells with an amine-reactive, non-membrane permeable biotinylation reagent, lysed these cells and performed immunoprecipitation (IP) of the biotinylated proteins with streptavidin beads. Western blot analysis of this IP showed that there is a significant fraction of the total pool of TMED10 protein that localizes to the cell surface (online supplemental figure 2b). This result prompted us to consider that TMED10 and PD-1 engage in a physical interaction. By re-analyzing publicly available mass spectrometry data of IPs of OST-tagged PD-1,^76^ we found that in both human and murine T cells, TMED proteins interact with PD-1 (figure 2a, online supplemental figure 2c,d). We confirmed these findings in our own model by performing an IP for PD-1 showing TMED10 co-IP (online supplemental figure 2e). To address the involvement of vesicular transport in TMED10-dependent PD-1 regulation, we treated *Tmed10* WT or KO cells with brefeldin A (BrefA), a vesicular transport inhibitor. In WT cells, BrefA significantly reduced PD-1 abundance at the cell surface, as measured by flow cytometry (figure 2b,c). Conversely, the levels of PD-1 in *Tmed10* KO cells remained as low after treatment with BrefA as in untreated cells (figure 2b,c). Collectively, these data imply that TMED10, and potentially other TMED proteins, function as chaperones for PD-1 and that they promote its vesicular trafficking to the T cell surface.

We next asked whether this reduced stability of PD-1 on TMED10 inactivation would result in any functional changes in CD8 T cells. We analyzed resting and anti-CD3-activated WT and *Tmed10* KO cells by RNA sequencing. OT-I CD8 T cells express PD-L1 with which they can inhibit each other in *trans*.^77^,^79^ Our stimulation approach thus allowed us to assess the extent and effects of PD-1-mediated inhibition on T cell activation, without having to mix in a second, PD-L1-expressing cell type such as tumor cells. This monocellular source of RNA enhances analysis fidelity. In general, similar transcriptional signatures were induced in *Tmed10-*proficient and *Tmed10*-deficient cells on activation (online supplemental figure 2f), reinforcing the notion that cells lacking TMED10 can still be successfully activated (figure 1). However, a number of differentially expressed genes were identified between WT and *Tmed10* KO cells on activation (online supplemental figure 2g, online supplemental table 4). Gene Ontology (GO) term enrichment and clustering of the significant terms by semantic similarity^80^ revealed five main clusters separating WT and *Tmed10* KO cells transcriptionally: proliferation, immune cell function, metabolism, apoptosis and protein transport (figure 2d, online supplemental table 2). To understand the directionality of these differences, we performed Gene Set Enrichment Analysis (GSEA) using representative GO terms for each of the clusters. This showed enhanced transcriptional signatures of T cell proliferation and activation in *Tmed10* KO cells, as well as an enhanced signature of fatty acid oxidation, whereas no clear directionality could be found for the apoptotic signature (figure 2e,f and online supplemental figure 2h).

To functionally validate this enhanced activation signature of *Tmed10*-deficient T cells, we performed a co-culture of OT-I/Cas9 T cells with murine melanoma B16F10-OVA cells. To assess the extent of activation, we measured the concentration of released effector cytokines, interferon (IFN)-γ, tumor necrosis factor (TNF) and interleukin (IL)-2, in the culture medium after the engagement of tumor cells by the T cells. Additionally, to determine the involvement of PD-1 in the activation of WT and *Tmed10* KO cells, we treated cells with PD-1 antibody. We found that *Tmed10-*deficient CD8 T cells released more cytokines than their WT counterparts, corroborating our RNA sequencing data (figure 2g). Furthermore, PD-1 blockade significantly increased cytokine release in WT cells (figure 2g). This effect was not observed in *Tmed10* KO cells, implying that the increased activation observed in these cells was due to their reduced abundance of PD-1 (figure 2g). We obtained similar results when we activated sg*Tmed10* cells in monocultures, where PD-L1 expressed on the T cells themselves could likely affect other T cells in trans given their close proximity in these cultures (online supplemental figure 2i–l). Together, these mechanistic experiments indicate that the deletion of *Tmed10* allows for superior CD8 T cell effector function due to reduced levels of cell-surface PD-1. To extend these findings, we performed immunoblot analyses of cells harboring sgCtrl or sg*Tmed10* after short-term TCR triggering by CD3 antibody to assess activation-induced nuclear factor kappa B (NFκB) signaling events in the context of PD-L1. While sg*Tmed10* cells displayed enhanced phospho-NFκB p65 after activation, this was only partially dependent on their differential levels of PD-1, as evidenced by the partial rescue on the addition of anti-PD-1 (online supplemental figure 3a). This may imply that also other proteins affected by the loss of TMED10 could affect the perturbed T cells.

To further dissect and understand the molecular consequences of *Tmed10* deletion in CD8 T cells, we projected putative TMED10 regulation motifs on PD-1.^71^ Both motifs were found to be present and conserved in the amino acid sequence of PD-1 (online supplemental figure 3b,c). To expand on this, we next performed mass spectrometry of perturbed cells on activation with CD3 antibody. This analysis confirmed our findings made with the cytokine release assay, as cells lacking functional TMED10 expressed more IFN-γ (figure 2h). We also found that cells lacking TMED10 had reduced protein levels of several other TMED family proteins, including the hits from the genetic screen TMED2 and TMED9 (figure 2h and online supplemental table 3). This apparent interdependency between TMED proteins is consistent with previous observations, in particular for TMED10 and TMED2.^6881^,^84^ We confirmed and extended this finding by performing western blot analyses of cells lacking TMED2, TMED9 or TMED10. We found that the genetic inactivation of a single *Tmed* gene reduced the protein abundance of the others (figure 2i).

To better understand the role of TMED10 in regulating cell surface proteins, we performed mass spectrometry of isolated cell surface proteins for both sgCtrl and sg*Tmed10* cells (online supplemental table 3). To enrich for proteins directly regulated by TMED10, we selected from the proteins that were differentially abundant between these two conditions those that also carried the putative TMED10 regulation motifs. This analysis enabled us to identify four other proteins potentially regulated by TMED10 directly: CTLA-4, UNC93B1, CXCR4 and IGF2R (online supplemental figure 3d,e). We validated that *Tmed10* KO cells indeed displayed lower levels of CTLA-4 on their cell surface (online supplemental figure 3f).

### AGN192403 reduces PD-1 abundance and enhances CD8 T cell activity by lysosomally degrading PD-1-TMED10 complex

The interdependency between different TMED proteins offered a potential translational avenue, because a small molecule, AGN192403 (also known as BRD4780 and originally described as an I_1_-imidazoline receptor agonist), has been shown to bind to and reduce TMED9 protein levels.^85 86^ We hypothesized that, due to the interdependent nature of TMED protein abundance, AGN192403 could be used to reduce the abundance of multiple TMED proteins in CD8 T cells as a therapeutic strategy to suppress PD-1 abundance.

**Figure 3:**
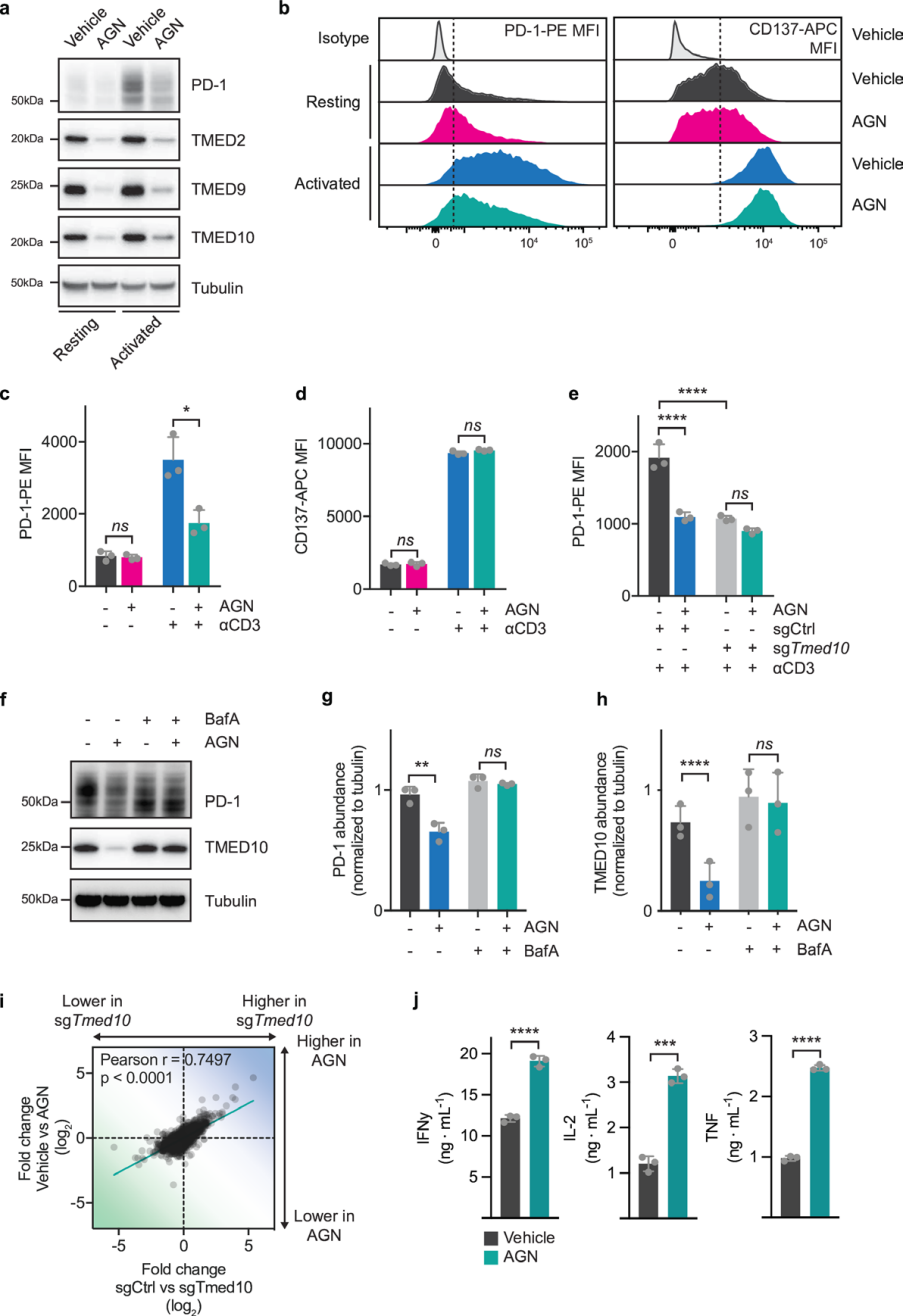
AGN192403 reduces PD-1 abundance and enhances CD8 T cell activity by lysosomally degrading PD-1-TMED10 complex. (**a**) Western blot analysis of PD-1, TMED10, TMED9 and TMED2 in OT-I/Cas9 CD8 T cells before or after activation with CD3 antibody for 24 hours in the presence or absence of AGN192403 (100 µM). The size markings indicate the size of the closest molecular weight marker. (**b**) Representative flow cytometry histograms of PD-1-PE (left) or CD137-APC (right) of resting or anti-CD3-activated OT-I/Cas9 CD8 T cells treated, or not, with AGN192403 (AGN; 100 µM) for 24 hours. (**c**) Quantification of samples in (b) for PD-1-PE abundance. Each data point indicates data obtained with CD8 T cells from an independent spleen. Error bars denote SD. Statistical analysis was performed with a one-way ANOVA, followed by a Tukey post hoc test. (**d**) As in (**c**) but for CD137-APC abundance. (**e**) Quantification of PD-1-PE abundance by flow cytometry for OT-I/Cas9 CD8 T cells carrying an sgRNA targeting *Tmed10* or a non-targeting control sgRNA after activation with CD3 antibody, in the presence or absence of AGN192403 (AGN; 100 µM). Each data point indicates data obtained with CD8 T cells from an independent spleen. Error bars denote SD. Statistical analysis was performed with a one-way ANOVA, followed by a Tukey post hoc test. (**f**) Western blot analysis of PD-1 and TMED10 abundance in OT-I/Cas9 CD8 T cells after activation with CD3 antibody for 24 hours in the presence or absence of AGN192403 (AGN; 100 µM) and/or bafilomycin A1 (100 nM). The size markings indicate the size of the closest molecular weight marker. (**g**) Quantification of experiment in (**f**) where PD-1 abundance was normalized to tubulin abundance. Each data point indicates data obtained with CD8 T cells from an independent spleen. Error bars denote SD. Statistical analysis was performed with an RM one-way ANOVA, pairing the data from independent experiments, followed by a Tukey post hoc test. (**h**) As in (**g**) but for TMED10 abundance. (**i**) Correlation plot of transcriptomic changes between activated OT-I/Cas9 CD8 T cells carrying a non-targeting control sgRNA and an sgRNA targeting *Tmed10* (x-axis) and activated OT-I/Cas9 CD8 T cells treated with vehicle or AGN192403 (AGN; 100 µM; y-axis). Each data point indicates the relative changes in the expression of a single gene. (**j**) Cytokine release of IFN-γ (left), IL-2 (middle) and TNF (right) as measured by cytometric bead array of OT-I/Cas9 CD8 T cells after co-culture with B16F10-OVA tumor cells in a 1:4 ratio, in the presence or absence of AGN192403 (AGN; 100 µM). Each data point indicates data obtained with CD8 T cells from an independent spleen. Error bars denote SD. Statistical analysis was performed with a Student’s t-test for each cytokine *p<0.05; **p<0.01; ***p<0.001; ****p<0.0001. ANOVA, analysis of variance; IFN, interferon; IL, interleukin; MFI, mean fluorescence intensity; mean fluorescence intensity; PD-1, programmed cell death protein-1; RM, repeated measures; TNF, tumor necrosis factor.

To test this, we treated CD8 T cells with AGN192403 before or after activation with CD3 antibody and performed western blotting and mass spectrometry. In both analyses, we observed indeed a substantial reduction in the abundance of a number of TMED proteins (figure 3a and online supplemental figure 4a). Correspondingly, PD-1 abundance in activated CD8 T cells was reduced on AGN192403 treatment (figure 3a). With a similar experimental set-up, we also determined PD-1 and CD137 abundance by flow cytometry and found that cells treated with AGN192403 had reduced PD-1 abundance, yet maintained CD137 abundance, mirroring our findings with genetic inactivation of *Tmed10* (figure 3b–d). Furthermore, we obtained similar results using human CD8 T cells (online supplemental figure 4b–e).

Given that AGN192403 is an imidazoline receptor ligand, it was important to determine to what extent TMED10 was required for its effects in CD8 T cells. We performed an epistatic experiment, in which either sgCtrl or sg*Tmed10* CD8 T cells were treated with AGN192403 and analyzed for PD-1 abundance by flow cytometry. Whereas AGN192403 significantly reduced PD-1 levels in WT cells, it failed to further reduce PD-1 levels in *Tmed10* deficient cells, implying that TMED10 is required for the effect of AGN192403 on PD-1 abundance (figure 3e).

We next aimed to understand how AGN192403 treatment results in reduced PD-1 levels. It was shown before that AGN192403 treatment can result in the lysosomal degradation of TMED client proteins.^86^ To determine whether AGN192403 treatment can induce the lysosomal degradation of PD-1 in CD8 T cells, we treated cells during anti-CD3 activation with an inhibitor of lysosomal degradation, bafilomycin A1 (BafA), in the presence or absence of AGN192403 and analyzed PD-1 and TMED10 abundance by western blotting. BafA co-treatment not only rescued the reduction of PD-1 abundance by AGN192403, but also that of TMED10 (figure 3f–i). These mechanistic experiments together imply that AGN192403 treatment results in the TMED10-dependent, lysosomal degradation of PD-1.

We next investigated whether AGN192403 treatment mimics the functional effects that we had observed with the genetic inactivation of *Tmed10*. We performed RNA sequencing of vehicle-treated or AGN192403-treated CD8 T cells before or after CD3 activation. A comparison of the differences showed a good correlation between genetic and pharmacological perturbation (figure 3i, online supplemental table 4). We validated this finding by comparing significantly differentially expressed transcripts between vehicle-treated and AGN192403-treated cells to those found between sgCtrl and sg*Tmed10* cells, all after activation. We found that AGN192403 treatment affected the large majority of the differentially expressed transcripts observed after knocking out *Tmed10* (online supplemental figure 4f). To assess the functional similarity between AGN192403 treatment and *Tmed10* KO, we performed a cytokine release assay after co-culture with B16F10-OVA cells. Similar to what was observed for genetic inactivation of *Tmed10*, AGN192403 treatment caused enhanced cytokine release by CD8 T cells (figures2g  3j). This was also true for monocultures of T cells treated with AGN192403 (online supplemental figure 4g). AGN192403 also reduced CTLA-4 abundance in these cells, mirroring our findings made with the KO of *Tmed10* (online supplemental figure 4h). Collectively, these data show that AGN192403 treatment pharmacologically mimics the effect of *Tmed10* genetic deletion by driving the lysosomal degradation of the PD-1-TMED10 complex, resulting in enhanced T cell activation.

### TMED expression in CD8 T cells positively correlates with T cell dysfunction and lack of immunotherapy response in patients

**Figure 4:**
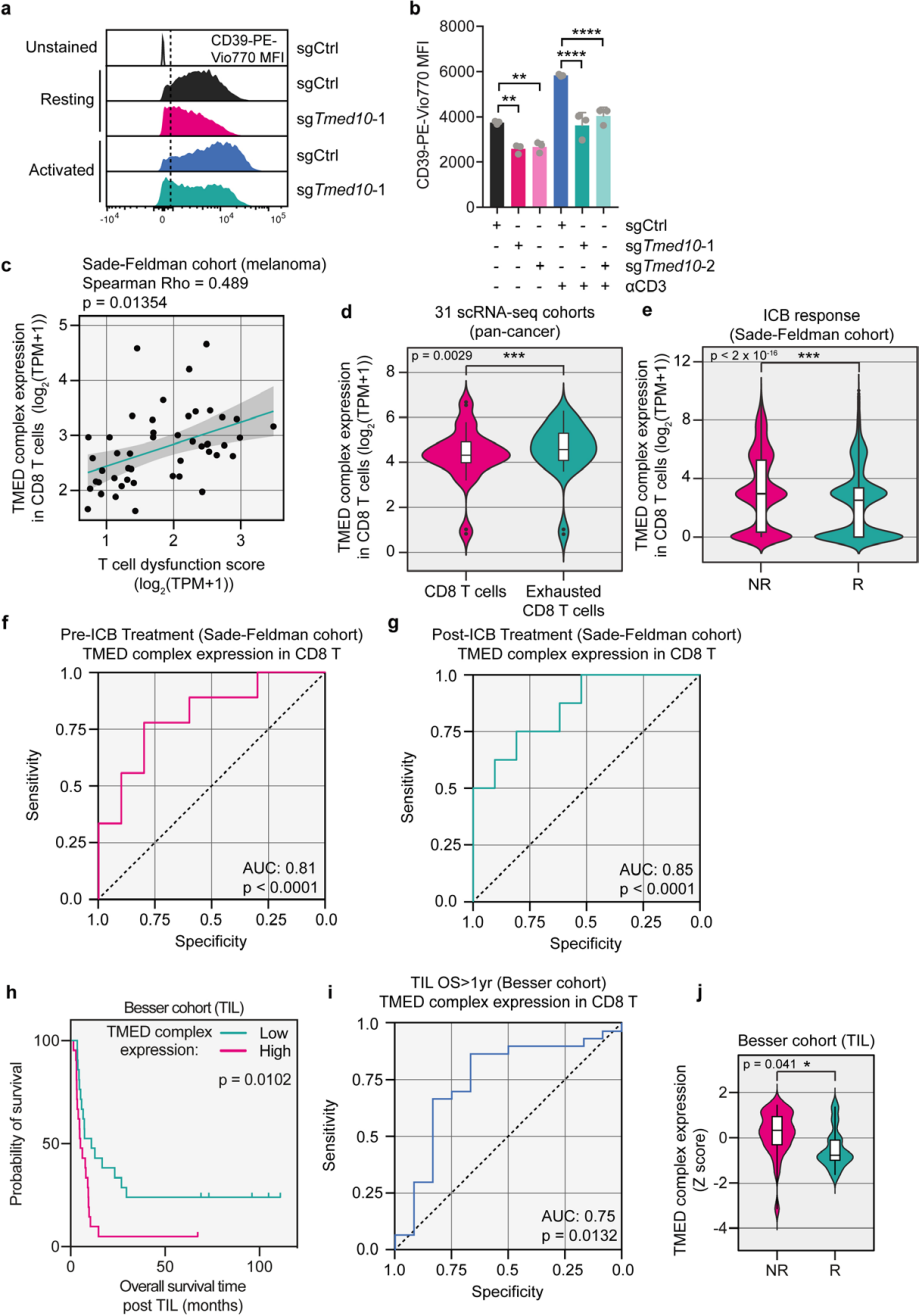
TMED expression in CD8 T cells positively correlates with T cell dysfunction and lack of immunotherapy response in patients. (**a**) Representative flow cytometry histograms of CD39-PE-Vio770 MFI of resting or anti-CD3-activated OT-I/Cas9 CD8 T cells carrying either an sgRNA targeting *Tmed10* or a non-targeting control sgRNA. (**b**) Quantification of samples in (**a**) for CD39-PE-Vio770 abundance. Each data point indicates data obtained with CD8 T cells from an independent spleen. Error bars denote SD. Statistical analysis was performed with a one-way analysis of variance, followed by a Tukey post hoc test. (**c**) Correlation plot of TMED complex expression and T cell dysfunction signature expression in CD8 T cells in melanomas from patients.^12^ Each data point indicates mean expression across all CD8 T cells in an individual patient. The best fit line is shown with the shaded region denoting the 95% CI. (**d**) TMED complex expression in exhausted CD8 T cells and other CD8 T cells in 31 single-cell RNA sequencing datasets. Each data point indicates the mean expression of the TMED complex across all exhausted CD8 T cells or all CD8 T cells in each dataset. In the boxplots, the center line, box edges and whiskers denote the median, IQR and the rest of the distribution respectively, with outliers being shown separately. Statistical testing was performed by the Wilcoxon rank-sum test. (**e**) TMED complex expression in CD8 T cells of responders (R) and non-responders (NR) to ICB, respectively.^12^ Each data point indicates expression of the TMED complex in single CD8 T cells. In the boxplots, the center line, box edges and whiskers denote the median, IQR and the rest of the distribution, respectively, with outliers being shown separately. Statistical testing was performed by the Wilcoxon rank-sum test. (**f**) ROC curve analysis of patients from the Sade-Feldman cohort who had their tumor biopsied before the start of ICB treatment, using TMED complex expression to distinguish R from NR patients. (**g**) As in (**f**) but for patients whose biopsy was taken after the onset of therapy. (**h**) Kaplan-Meier survival curve of patients who received TIL therapy with a TIL product with high (50% highest expressors) or low (50% lowest expressors) expression of the TMED complex. Statistical testing was performed by log-rank test. (**i**) ROC curve analysis of patients from the TIL cohort, using TMED complex expression in the TIL product to distinguish patients who failed to survive for more than 1 year from those who did. (**j**) TMED complex expression in patients who survived for more than 1 year after TIL infusion (R) and those who did not (NR). In the box plots, the center line, box edges and whiskers denote the median, IQR and the rest of the distribution, respectively, with outliers being shown separately. Statistical testing was performed by Student’s t-test. *p<0.05; **p<0.01; ***p<0.001; ****p<0.0001. ICB, immune checkpoint blockade; MFI, mean fluorescence intensity; ROC, receiver-operating characteristic; TIL, tumor-infiltrating lymphocytes; AUC, area under the ROC curve.

Having shown that TMED10 is a critical regulator of PD-1 abundance, and with the knowledge that PD-1 can enforce T cell exhaustion,^8 14 26^ we next investigated a possible role for TMED10 in governing T cell dysfunction. We first analyzed WT or *Tmed10* KO cells by flow cytometry to assess their abundance of CD39, a hallmark surface receptor for T cell dysfunction.^21 22^ Both before and after CD3 activation, CD39 abundance was significantly lower on CD8 T cells lacking functional TMED10 (figure 4a,b and online supplemental figure 5a–c). This finding, together with the observations that *Tmed10* KO cells have increased effector function (figure 2), suggests a role for TMED proteins in regulating T cell dysfunction.

To expand on this assertion, we investigated the role of TMED proteins in patient tumors. We chose to look at several TMED genes rather than TMED10 only, since (1) we show that three TMED family genes (2, 9, 10) scored as significant hits in the genetic screen (figure 1b); (2) we validated that all three multiple TMED proteins regulate PD-1 abundance (figure 1f,h); (3) we show that expression of TMED proteins is dependent on one another (figure 2h,i); and (4) because of the intrinsic challenge of analyzing single genes in single-cell RNA (scRNA) sequencing data, studying gene sets reduces noise. We refer to the combination of TMED2, 9 and 10 as TMED complex. To query the role of the TMED complex in CD8 T cells, we made use of scRNA sequencing data of tumor-infiltrating lymphocytes (TIL) of a cohort of patients with melanoma treated with ICB.^12^ Using these data, we found a significant positive correlation between the expression of the TMED complex in CD8 T cells and a T cell dysfunction signature (figure 4c; online supplemental table 4).^12^ We expanded this analysis to 31 publicly available scRNA sequencing cohorts across multiple cancer indications. Consistent with the correlation above, we found that TMED complex expression was higher in exhausted CD8 T cells than in conventional CD8 T cells (figure 4d; online supplemental table 4).

Given this correlation between TMED complex expression and T cell dysfunction, we next asked whether TMED has any predictive power for immunotherapy outcome. We again analyzed the melanoma ICB cohort^12^ and found that in patients who failed to respond to their therapy, TMED complex expression in CD8 T cells was significantly higher than in those who did respond, irrespective of the time of biopsy and type of ICB (figure 4e and online supplemental figure 5d). No matching protein level information on PD-1 and/or TMED complex was available, precluding analysis of direct relationships in these datasets. Extending these observations, receiver-operating characteristic (ROC) analysis revealed that TMED complex expression in CD8 T cells accurately discriminated responding from non-responding patients, irrespective of whether the analyzed biopsy was taken before or after therapy onset (figure 4f,g). We found similar results in an independent dataset^18^ (online supplemental figure 5e,f).

To further clinically corroborate the relevance of TMED complex expression in immunotherapy, we continued by analyzing a cohort of patients who underwent TIL therapy. Before the expanded TIL product was infused into the patient, a sample was taken for RNA sequencing. As TIL products comprise mostly T cells,^87^ this allowed us to assess the T cell-intrinsic expression of the TMED complex (online supplemental table 4). These data, together with the knowledge of the response to TIL therapy in these patients, allow us to correlate TMED expression to the clinical outcome of this type of immunotherapy, thus complementing our scRNA sequencing data of CD8 T cells. We found this metric to have prognostic power, because patients who received a TIL product with high expression of the TMED complex had a shorter overall survival than those who had received a TIL product with low TMED complex expression (figure 4h). By ROC analysis, we also found that TMED complex expression in the TIL infusion product could accurately identify patients who survive more than 1 year after receiving TIL therapy (figure 4i). Additionally, the TIL infusion products of patients who responded to their treatment had lower expression of the TMED complex than the TIL products of patients who did not (figure 4j). Together, these clinical data support our results in cultured T cells and highlight a potential role for TMED proteins in regulating human T cell dysfunction in patients, thereby influencing clinical responses to two different types of immunotherapies.

### AGN192403 overcomes dysfunction in tumor-infiltrating CD8 T cells

**Figure 5:**
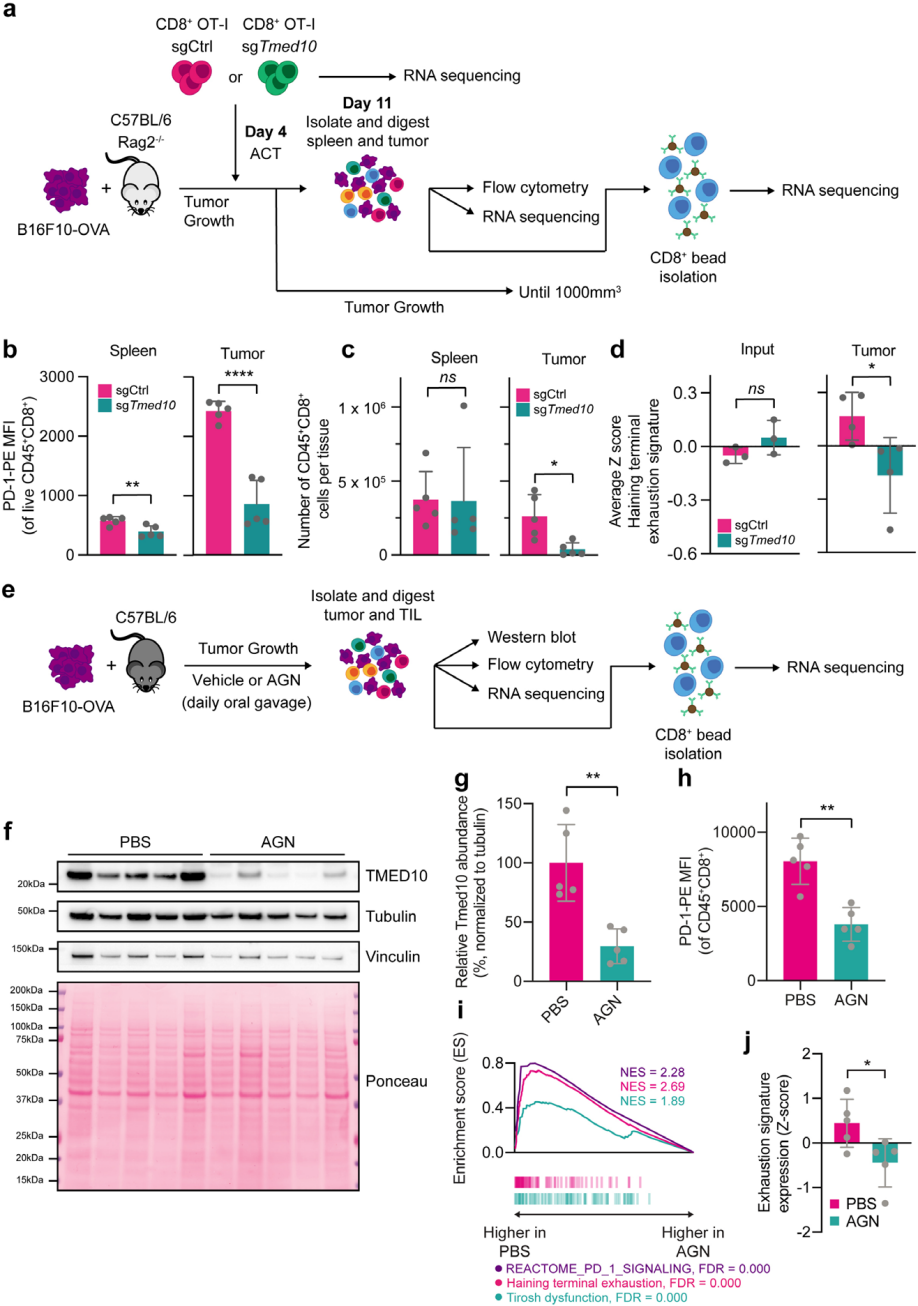
AGN192403 overcomes dysfunction in tumor-infiltrating CD8 T cells. (**a**) Schematic representation of ACT experiment and downstream analyses. (**b**) Quantification of PD-1-PE abundance by flow cytometry of tumor-infiltrating CD8 T cells, identified as live, CD45^+^CD8^+^, from spleens and tumors from mice that received ACT with sgCtrl or sg*Tmed10* CD8 T cells. Each data point indicates data obtained with an independent tumor. Error bars denote SD. Statistical analysis was performed with a Student’s t-test for each tissue. (**c**) Flow cytometry-based quantification of live CD45^+^CD8^+^ cells in spleens and tumors from mice in b. Each data point indicates data obtained with an independent tumor. Error bars denote SD. Statistical analysis was performed with a Student’s t-test. (**d**) The average Z-score expression of the terminal exhaustion signature of the mice in b. Each data point indicates the average signature expression in the CD8^+^ TIL population of an individual mouse. Error bars denote SD. Statistical analysis was performed with a Student’s t-test for each tissue. (**e**) Schematic representation of animal experiment and downstream analyses. (**f**) Western blot analysis of PD-1 and TMED10 in individual tumors treated with PBS or AGN192403 (AGN; 125 mg/kg). The size markings indicate the size of the closest molecular weight marker. (**g**) Quantification of relative TMED10 abundance of the samples in f. Each data point indicates data obtained with an independent tumor, normalized to tubulin abundance. Error bars denote SD. Statistical analysis was performed with a Student’s t-test. (**h**) Quantification of PD-1-PE abundance by flow cytometry of tumor-infiltrating CD8 T cells, identified as live, CD45^+^CD8^+^, from tumors from mice treated with PBS or AGN192403 (AGN; 125 mg/kg). Each data point indicates data obtained with an independent tumor. Error bars denote SD. Statistical analysis was performed with a Student’s t-test. (**i**) Gene Set Enrichment Analysis plots of the gene sets REACTOME_PD_1_SIGNALING, Haining terminal exhaustion and Tirosh dysfunction,^13 20^ comparing CD8^+^ TIL from animals treated with PBS to those from animals treated with AGN192403 (AGN; 125 mg/kg). (**j**)^13 20^ The average Z-score expression of the terminal exhaustion signature. Each data point indicates the average signature expression in the CD8^+^ TIL population of an individual mouse. Error bars denote SD. Statistical analysis was performed with a Student’s t-test. *p<0.05; **p<0.01; ***p<0.001; ****p<0.0001. ACT, adoptive cell transfer; MFI, mean fluorescence intensity; PBS, phosphate-buffered saline; PD-1, programmed cell death protein-1; OVA, ovalbumin; TIL, tumor-infiltrating lymphocytes.

Given this positive correlation between TMED complex expression and T cell dysfunction in patient tumors, we next examined whether CD8 T cells lacking TMED10 were more efficacious in combating tumors than their WT counterparts. For this, we performed adoptive cell transfer with either sgCtrl or sg*Tmed10* CD8 T cells in *Rag2*^−/−^ mice carrying B16F10-OVA tumors (figure 5a). Seven days after adoptive cell transfer (ACT), we analyzed the spleens and tumors of sentinel animals by flow cytometry. As in our in vitro experiments, CD8 T cells carrying sg*Tmed10* displayed significantly less PD-1 on their cell surface, while CD137 levels remained unchanged (figure 5b, online supplemental figure 6a). We also determined the impact of perturbation of *Tmed10* on T cell dysfunction in vivo, by measuring the fraction of CD8^+^CD39^+^PD-1^+^ cells.^21 22^ Fewer double positive cells were present in the cells lacking functional TMED10, implying a more fit T cell repertoire (online supplemental figure 6b). However, and importantly, when we quantified the number of CD8 T cells present in the spleen and tumor, we observed much fewer *Tmed10* KO T cells had infiltrated into tumors than WT T cells (figure 5c).

As the general fitness of the cells harboring sg*Tmed10* did not markedly differ from their WT counterparts, as evidenced by the similar number of cells present in the spleen, we hypothesized that this defect may stem instead from impaired homing to, or retention within, the tumor tissue. To test this hypothesis, we performed RNA sequencing of the whole tumors in the sentinel animals. Additionally, we purified CD8^+^ cells from these tumors and analyzed these by RNA sequencing also, with T cells that were harvested before ACT serving as controls (figure 5a, online supplemental table 4). The analysis of the whole tumor digest confirmed the observed relatively low abundance of *Tmed10* KO CD8 T cells from the tumor microenvironment (TME; online supplemental figure 6c).

To assess potential defects in migration, we measured the chemokines expressed in the B16F10-OVA tumors (online supplemental figure 6d). For those chemokines expressed, we then queried whether receptors for these chemokines were differentially expressed in sgCtrl and sg*Tmed10* CD8 T cells using our CD8 T cell-enriched RNA sequencing. The only chemokine receptor that was differentially expressed in this analysis was *Cxcr6* (online supplemental figure 6e). This protein acts as a receptor for CXCL16, and has been implicated in the migration to, and local maintenance of, CD8 T cells within tumors.^88 89^ This gene was already deregulated at the RNA level before ACT (online supplemental figure 6f).

Importantly, the *Tmed10* KO cells that were able to successfully reside in the tumor were generally fitter, as evidenced by their reduced expression of an exhaustion signature (figure 5d). These fewer, but fitter cells, could equally affect tumor growth as WT T cells, as evidenced by similar cytokine response signatures and similar tumor volume measurements after ACT (online supplemental figure 6g,h).

This result prompted us to determine whether acute depletion of TMED10 in CD8 T cells already present in the TME could enhance CD8 T cell fitness and antitumor immunity. We thus assessed whether the pharmacological degradation of TMED proteins by AGN192403 could overcome or prevent the T cell dysfunction often seen in murine tumor models. We injected B16F10-OVA cells into syngeneic C57BL/6 mice, and on tumor establishment administered vehicle or AGN192403 by daily oral gavage for a total of 6 days. The day after the final treatment, we harvested sentinel mice to assess the quality of the immune infiltrate in the tumors (figure 5e). Western blotting of these tumors showed that AGN192403 treatment had successfully reduced TMED10 abundance in vivo (figure 5f,g). Moreover, flow cytometry of these tumors showed that PD-1 abundance on CD8 T cells was substantially reduced in mice treated with AGN192403, while CD137 abundance remained similar (figure 5h, online supplemental figure 7a), recapitulating our in vitro observations. Additionally, we found that compared with vehicle-treated mice, mice receiving AGN192403 had significantly fewer CD8^+^CD39^+^PD-1^+^ cells, indicating a T cell repertoire that is associated with greater fitness (online supplemental figure 7b,c). We found that in tumors of AGN192403-treated mice, T cells experienced less inhibitory PD-1 signaling, consistent with the observed reduction in PD-1 levels (figure 5i). Additionally, we observed a reduced level of T cell dysfunction and/or exhaustion in these cells, as measured by two independent gene sets of T cell dysfunction (figure 5i,j, online supplemental figure 7d–f, online supplemental table 4),^13 20^ implying that AGN192403 treatment can overcome T cell dysfunction in vivo. In line with these findings, we also observed a significant antitumor effect of AGN192403 treatment (online supplemental figure 7g–i).

## Discussion

We report on an FACS-based genomic screen for specific and critical regulators of PD-1 expressed on the surface of primary CD8 T cells. Top hits included several members of the TMED family of chaperones, the inactivation of which (either genetically or pharmacologically) led to the reversion of T cell dysfunction in cultured T cells and in mice. Our CD137/PD-1 dual-marker, sort-based screen was designed to identify regulators of PD-1 specifically, rather than factors required for general T cell activation. The TMED proteins, in particular TMED2, TMED9 and TMED10, fulfilled this criterion: their perturbation led to a reduction of PD-1 protein levels, did not prevent T cell activation but instead overcame exhaustion.

We demonstrate that by functioning as chaperones, mediating the optimal delivery of PD-1 to the cell surface, the TMED proteins limit CD8 T cell activity. Clinically corroborating these results, in patient tumors we found a positive correlation between T cell dysfunction, lack of immunotherapy response and TMED expression in CD8 T cells. We confirmed a causal role for the TMED proteins in establishing T cell exhaustion also in a murine melanoma model, in which TMED inhibition by the small molecule AGN192403 led to a reversal of T cell exhaustion. We established this both in terms of the number of CD39^+^PD1^+^ cells among the CD8^+^ T cell repertoire of TIL, and more globally by assessing the expression of two separate exhaustion signatures in CD8^+^ TIL. Together, these data indicate that the levels of TMED proteins in CD8 T cells may be good indicators of the extent of PD-1-induced exhaustion.

Our fundamental understanding of the mechanism of PD-1 expression, stability and localization is still incomplete. Through our FACS-based genetic screen, we identified the TMED proteins as novel regulators of PD-1 abundance that are involved in a previously underappreciated aspect of PD-1 regulation: its trafficking towards the cell surface. Importantly, this regulation of PD-1 is uncoupled from the activation status of CD8 T cells. The only other, activation-independent regulator of PD-1 abundance known so far is FBXO38, which regulates the proteasomal turnover of PD-1.^90^ Aside from PD-1, the TMED proteins have also been reported to regulate the vesicular transport of a number of other client proteins, including IL-1 family members, STING, and MUC1-fs.^71 86 91 92^ While proteins regulated by the TMED family are seemingly diverse, some commonalities between their client proteins have been observed. For example, TMED proteins regulate the turnover of misfolded proteins during ER stress through a pathway termed RESET (rapid ER stress-induced export).^70^ During RESET, misfolded, GPI-anchored proteins are exported from the ER by TMED10 to transiently access the cell surface before being degraded in the lysosome; akin to the mechanism by which we demonstrate that AGN192403 degrades the TMED10-PD-1 complex. TMED10 is also involved in unconventional protein secretion, which allows for leaderless proteins to be secreted in a manner that bypasses ER-Golgi trafficking.^71^ However, PD-1 is a single-pass, type 1 transmembrane protein and contains a signal peptide. Therefore, it does not seem to represent a canonical RESET or UPS client, but instead would signify a novel type of TMED client.^93 94^ Total PD-1 levels may also be affected by the loss of TMED10, pointing to a complex mechanism of PD-1 regulation by TMED10, possibly involving also secondary effects AGN192403 treatment likely acts through a different mechanism. TMED proteins may play important roles in shuttling transmembrane proteins to the cell surface during periods of ER stress, as was recently shown for other clients,^95^ and as can be expected during T cell activation.^96^

We show that PD-1 is not regulated by a single TMED protein only: we identified TMED2, TMED9 and TMED10 to all regulate PD-1 abundance. We also observed an interdependency of the different TMED proteins: both the KO of TMED10 and treatment with AGN192403 led to the loss of multiple TMED proteins, confirming earlier reports, although the underlying mechanism remains unclear.^68 74 82 84^ This interdependency makes it difficult to assert which of the TMED proteins directly interacts with PD-1. While we do show an interaction between TMED10 and PD-1, this may be indirect, potentially through an interactive complex comprising multiple TMED proteins, similar to other contexts.^68 82^

The observation that TMED proteins regulate PD-1 abundance, and in particular the ability to target them pharmacologically, may come with translational potential. Whereas PD-1 activity can be limited by small molecule inhibitors, specifically by preventing the interaction between PD-1 and PD-L1,^97^ AGN192403 therapy would constitute a novel way to target PD-1, namely by inducing its lysosomal degradation. Nivolumab and pembrolizumab show a long half-life and high receptor occupancy, independent of the dosage administered.^98^,^100^ Additionally, as with most antibody-based therapeutics, poor tumor penetration and oral bioavailability may limit optimal therapy efficacy, while high production costs may limit therapy adoption.^101^ While it seems unlikely that antibody-based PD-1 therapy will be replaced in the near future, we speculate that TMED inhibitors could be used to control PD-1 activity with more precision, acting as rheostats for PD-1. In this respect, it is noteworthy that TMED inhibition causes downregulation not only of PD-1 but also of CTLA-4, which could add to its therapeutic utility. For any future TMED inhibitor it will be necessary to determine the specificity for CD8 T cells and potential effect on other cells. Furthermore, we observed that on ACT in mice carrying B16F10-OVA tumors, *Tmed10* KO T cells were much less capable of infiltrating tumors than WT T cells, potentially through the downregulation of CXCR6. While it is currently unclear whether this migration defect manifests also in other tumor models and/or in patient tumors, our data suggest that a TMED inhibitor acting on TIL would be a preferable therapeutic strategy, rather than the adoptive transfer of TMED KO T cells. The potential role of CXCR6 in T cell migration as a function of TMED modulation merits further investigation.

Our data imply that by merely partially limiting the abundance of PD-1, CD8 T cell functionality may be boosted in patient tumors by strongly enhancing T cell fitness. The observation that moderately reducing PD-1 levels on CD8 T cells is sufficient to promote T cell fitness seems to follow a Goldilocks principle, in which the reduced PD-1 abundance allows for the direct alleviation of inhibition of T cell activity, while the same level of PD-1 abundance is sufficient to protect the polyfunctional T cell repertoire from activation-induced cell death,^8 14 26 29 33 41^ but future investigations should ascertain the validity of such a model (online supplemental figure 8). This notwithstanding, our finding that the TMED family regulates PD-1 cell surface abundance, in combination with its amenability to small molecule intervention, offers not only fundamental insight into the mechanism governing the activity of a key inhibitory receptor in immunotherapy, but may also provide a translational opportunity.

## Methods

### Materials availability

Information and materials can be obtained from the lead contact, Daniel S Peeper, on reasonable request.

### Data and code availability

The RNA sequencing data presented in this article can be accessed via theNational Center for Biotechnology Information (NCBI) Gene Expression Omnibus (GEO) database using accession numbers GSE161211 and GSE252956GSE252956. Due to the extent to which patient consent was given, only the respective expression values, and not the full sequencing data, were deposited for the TIL cohort (online supplemental table 4). The mass spectrometry data have been deposited to the ProteomeXchange Consortium via the PRIDE partner repository with the dataset identifier PXD026984. General code can be found on the Peeper laboratory github (https://github.com/PeeperLab/Rtoolbox); specific code can be requested from the lead contact.

### Experimental model and subject details

#### Cell lines, primary cultures and human participants

The B16F10 cell line (male) was obtained from American Type Culture Collection (ATCC) and transduced with a lentiviral construct encoding for the full-length OVA protein.^102^ The Platinum-E cell line (female) was obtained from the internal Peeper laboratory stock. Both cell lines were routinely confirmed to be *Mycoplasma*-negative by PCR.^103^ They were maintained in Dulbecco’s Modified Eagle Medium (DMEM), containing 9% fetal bovine serum (FBS; Sigma), penicillin (100 U/mL, Gibco) and streptomycin (100 µg/mL, Gibco). Human CD8 T cells were isolated from healthy donor buffycoats (Sanquin) of both male and female anonymous donors, who provided written informed consent approved by the Dutch Ministry of Health. These cells were maintained in Roswell Park Memorial Institute Medium (RPMI) with 9% FBS, penicillin (100 U/mL), streptomycin (100 µg/mL) and 100 U/mL human IL-2 (Proleukin, Novartis). Murine CD8 T cells were isolated from the spleens of both male and female OT-I/Cas9 mice. These cells were maintained in RPMI with 9% FBS, penicillin (100 U/mL), streptomycin (100 µg/mL), 2-Mercaptoethanol (50 µM, Merck), murine IL-2 (10 ng/mL, ImmunoTools), murine IL-7 (0.5 ng/mL, ImmunoTools) and murine IL-15 (1 ng/mL, ImmunoTools). For TIL analysis, RNA of infused TIL products from both male and female, patients with stage IV melanoma was used^87^ (NCT00287131). These patients signed an informed consent approved by the Israeli Ministry of Health (Helsinki approval no. 3518/2004). Due to the extent to which patient consent was given, only the respective expression values, and not the full sequencing data, were deposited. For PD-L1 induction on B16F10-OVA cells, 1.2E5 tumor cells were seeded in 12-well plates and treated with 25 ng/mL IFN-γ for 24 hours (PeproTech). Cells were harvested by trypsinization (Thermo Fisher) prior to fluorescent staining and flow cytometric analysis as per below.

#### In vivo animal studies

All animal studies were approved by the animal ethics committee of the Netherlands Cancer Institute (NKI) and performed under approved NKI CCD14 (Centrale Commissie Dierproeven) according to the ethical and procedural guidelines established by the NKI and Dutch legislation. Mice were housed in single-use standard cages at controlled filtered air humidity (55%), temperature (21°C) and light cycle. All housing material, food and water were autoclaved or irradiated before use. Female, C57BL/6 (Janvier) mice were used between the ages of 8–12 weeks for animal experiments. For ACT experiments, female, *Rag2*^–/–^ (Janvier) animals were used. To generate OT-I/Cas9 mice, OT-I mice (The Jackson Laboratory) were crossed with Cas9-EGFP mice (The Jackson Laboratory) and subsequently backcrossed for at least 10 generations. Genotypes for both OT-I and Cas9 were confirmed homozygous by PCR as specified by the supplier. Spleens used for CD8 T cell isolation were harvested from OT-I/Cas9 mice with a maximum age of 12 months.

### Method details

#### Isolation, maintenance and treatment of splenic murine CD8 T cells

Spleens were harvested from OT-I/Cas9 mice, mashed subsequently through 100 µm and 70 µm cell strainers (Corning) and washed (addition of buffer, followed by centrifugation at 1,000× g for 5 min) in phosphate-buffered saline (PBS) (Gibco) containing 0.1% bovine serum albumin (BSA, Sigma; isolation buffer) before being resuspended and incubated in red blood cell lysis buffer (155 mM NH_4_Cl, 10 mM NaHCO_3_, 0.1 mM EDTA in distilled water; all Sigma) for 5 min. Splenocytes were then washed twice (once in PBS, once in isolation buffer) and resuspended in 1 mL isolation buffer. CD8 T cells were then isolated from this cell suspension using the Dynabeads Untouched Mouse CD8 Cells Kit (Thermo Fisher Scientific) following manufacturer’s instructions. Briefly, 100 µL antibody mix and 100 µL FBS was added to the cell suspension and incubated for 20 min on a rotator at 4°C. The cells were then washed in isolation buffer, resuspended in 2 mL isolation buffer and 1 mL pre-washed Dynabeads was added and the suspension was incubated for 15 min on a rotator at room temperature (21°C). This cell suspension was then passed over a magnet, and the non-bound fraction was harvested. The CD8 T cells that were obtained were then resuspended in medium and activated on non-tissue culture-treated 24-well plates (Corning) that were pre-coated with anti-CD3 (0.25 µg per well, Thermo Fisher Scientific) and anti-CD28 antibodies (2.5 µg per well, Thermo Fisher Scientific). After 48 hours, cells were resuspended at a concentration of 1 million cells per milliliter in medium and either retrovirally transduced, or maintained at this concentration by daily replacement of culture medium for at least 10 days until further analysis. In most experiments, cells were either left resting or were activated. To do so, after 10 days cells were grown on 24-well non-tissue culture-treated plates that were pre-coated with anti-CD3 antibody (for activated cells, 1.25 µg per well) or that were left untreated (for resting cells). In select experiments, cells were treated with BrefA for 24 hours (200 ng/mL, Invivogen). In select experiments, cells were treated with BafA for 24 hours (100 nM, Invivogen). In select experiments, cells were treated with AGN192403 (also known as BRD4780) for 24 hours (100 µM, Tocris)

#### Retroviral sgRNA vector and retroviral library construction

For the generation of a retroviral sgRNA vector, a geneblock (Integrated DNA Technologies) with the sgRNA cassette of lentiCRISPR V.2 (Addgene #52961) where the BsmBI sites were replaced by BbsI sites was cloned into the pMSCVpuro backbone (Clontech) by standard molecular techniques. To insert single sgRNA sequences into this backbone, gene-specific Cas9 target sequences were predicted using CHOPCHOP^104^ and cloned into the pMSCVpuro-sgRNA backbone by Golden-Gate cloning.^105^ For retroviral library construction, the sgRNA cassette of the Brie library (Addgene) was amplified by PCR and cloned into the pMSCVpuro backbone by standard molecular techniques. The integrity of the retroviral library was confirmed by deep sequencing (online supplemental table 1).

#### Retrovirus production

For retrovirus production, 3 million Platinum-E cells were seeded in a 10 cm dish (Greiner). After 24 hours, these cells were transfected by polyethyleneimine (45 µg/10 µg DNA, Sigma) with 5 µg of pCL-ECO (Addgene) plasmid and 5 µg of the transfer vector. After another 24 hours, the medium was replaced by Opti-MEM (Thermo Fisher Scientific) containing 2% FBS, penicillin (100 U/mL) and streptomycin (100 µg/mL). After a further 24 hours, the supernatant containing retrovirus was harvested, filtered through a 0.45 µm filter and stored at 4°C. Fresh medium was added to Platinum-E cells. The next day, the supernatant was again harvested and filtered, combined with the supernatant of the first harvest and concentrated 10 times by spin-filter centrifugation (100 kDa pore size, Merck). The concentrated supernatant was snap-frozen and stored at −80°C until use.

#### CD8 T cell transduction and selection

One million preactivated OT-I/Cas9 CD8 T cells were mixed with 1 mL concentrated retroviral supernatant in a non-tissue culture-treated 24-well plate well (Corning) that was pre-coated with RetroNectin (25 µg/well, Takara). The plate was then centrifuged at 3,000× g in a centrifuge with minimum acceleration and no brake. After centrifugation, the plate was placed in the incubator. The next day, cells were resuspended at a concentration of 1 million cells per milliliter in the medium. 24 hours later, the cells were put on puromycin selection (4 µg/mL, Sigma) and maintained for at least 8 days until analysis.

#### Flow cytometry

0.3 million cells per sample were spun down in 96-well V bottom plates (Brand) and washed in a 0.1% BSA in PBS solution (FACS buffer). Antibodies against markers of interest were then diluted in FACS buffer following manufacturer’s instructions, for a staining volume of 50 µL per well, or 100 µL for in vivo tumor samples. The cells were stained on ice for 30 min, protected from light. After incubation, cells were washed twice in FACS buffer and resuspended in FACS buffer before being analyzed on either an LSRFortessa Flow Cytometer or an LSR II Flow Cytometer (both BD). Dead cells were identified through the use of DAPI (BD) or the LIVE/DEAD Fixable Near-IR stain (Thermo Fisher Scientific). Antibodies against murine and human PD-1 (both PE-conjugated), murine and human CD137 (both APC-conjugated), murine CD8a (FITC-conjugated), murine CD39 (PE-Vio770-conjugated), murine CD45 (APC-Vio770-conjugated; all Miltenyi Biotec), murine CTLA-4 (PE-Cy7-conjugated, BioLegend), murine PD-L1 (BV711-conjugated, BD) human CD8A (BB515-conjugated, BD) and human IgG Fc (PE-conjugated, BioLegend). Isotype antibodies for Rat IgG2a (PE-conjugated) and Hamster IgG (APC-conjugated, both BioLegend) were used. In select experiments, a fusion protein consisting of the extracellular domain of PD-L1 and a human antibody Fc domain was used to label the cells (1 µg/well, BioLegend) for 1 hour on ice, before continuing with regular cell-surface staining.

#### Whole genome screen and analysis

300 million OT-I/Cas9 CD8 T cells were isolated, anti-CD3-activated, transduced with the retroviral Brie library and selected with puromycin for 7 days. After 2 days of puromycin selection, a library reference sample was taken (of 1,000× coverage). After 7 days of puromycin selection, a bulk sample was taken (of 1,000× coverage). On the seventh day, 2×10^9^ cells were re-activated. The next day, cells were harvested and stained with DAPI and antibodies targeting CD137 and PD-1. These cells were then sorted on FACSAria Fusion Cell Sorters (BD): DAPI^–^CD137^+^ cells were selected, and from that population, the top 10% PD-1 expressors and bottom 10% of PD-1 expressors were selected and sorted. At least 6×10^7^ cells were sorted for each arm of the screen. The screen was performed in duplicate, where each duplicate was performed with OT-I/Cas9 CD8 T cells from independent spleens. DNA was isolated from all populations by the Blood and Cell Culture DNA Maxi Kit (Qiagen) as per the manufacturer’s instructions. The sgRNA sequences present in the isolated DNA were amplified using the NEBNext High-Fidelity 2× PCR Master Mix (New England BioLabs) following manufacturer’s instructions, using the Brie_Fw and Brie_Rv primers (online supplemental table 5). The stretch of N nucleotides in the Brie_Fw primer denotes a unique DNA barcode used for each sample in the PCR. The amplicons generated were then analyzed on an Illumina HiSeq 2500 Sequencing system (Illumina). The identified sgRNA sequences were aligned to the Brie library, where reads with any mismatches were excluded from analysis. The resulting read count table (online supplemental table 1) was used as input for MAGeCK analysis (V.0.5.6),^63^ using the non-targeting sgRNA sequences as controls. To assess the relative depletion of essential genes, the library reference and bulk samples were used, and compared with known non-essential and core essential genes.^62^

#### Western blot

For western blot, cells were harvested, washed twice in PBS and lysed in RIPA buffer (50 mM TRIS pH 8.0, 150 mM NaCl, 1% Nonidet P40, 0.5% sodium deoxycholate, 0.1% SDS with Halt Protease and Phosphatase Inhibitor (Thermo Fisher Scientific)) for 30 min on ice. After incubation, the lysate was centrifuged for 10 min at 17,000× g and supernatant was harvested. Protein concentration was then measured by Bradford assay (Bio-Rad) and normalized. NuPAGE LDS Sample Buffer (Thermo Fisher Scientific) and 2-Mercaptoethanol (final concentration 2.5% v/v) was added and samples were boiled at 95°C in a heating block. Samples were then run on NuPAGE 4–12% Bis Tris gels (Thermo Fisher Scientific) using Precision Plus Protein Dual Color Standard (Bio-Rad) as a size indicator at 150 V for approximately 1 hour on ice. After electrophoresis, the protein was transferred to nitrocellulose membranes using the iBlot system (Thermo Fisher Scientific) following manufacturer’s instructions. After protein transfer, the membranes were incubated for 1 hour in blocking buffer (5% BSA, 0.2% Tween-20 in PBS). After blocking, membranes were incubated with primary antibodies, diluted in a blocking buffer, for 24 hours. At that point, membranes were washed three times in washing buffer (0.2% Tween-20 in PBS) for 5 min, after which the membranes were incubated with secondary antibodies for 1 hour. After this incubation, membranes were washed three times in washing buffer for 5 min before being developed using SuperSignal West Dura Extended Duration Substrate (Thermo Fisher Scientific), with images being captured on a ChemiDoc MP (Bio-Rad). Fiji (V.2.0.0) was used to quantify protein bands. Primary antibodies against mouse PD-1, human PD-1, Myc-tag, Vinculin (all Cell Signaling Technology), TMED2, TMED10 (both Santa Cruz), TMED9 and Tubulin (both Thermo Fisher Scientific), NFkB p65 (Santa Cruz Biotechnology), phospho-NFkB p65 (CST) and Vinculin (Sigma) and Cyclophilin B (CST) were used. Horseradish peroxidase (HRP)-conjugated, secondary antibodies against mouse and rabbit primary antibodies (both Thermo Fisher Scientific) were used to detect the primary antibodies. Ponceau S (Merck) was used to assess relative protein loading. Uncropped Western blot images can be found in online supplemental file 1.

#### Quantitative PCR

For qPCR, cells were harvested, washed twice in PBS and total RNA was isolated using the Isolate II RNA Mini Kit (Bioline) following manufacturer’s instructions. After isolation, 1 µg of RNA was transcribed into complementary DNA (cDNA) using the Maxima First Strand cDNA Kit (Thermo Fisher Scientific) according to manufacturer’s instructions. To perform qPCR, 0.5% of this cDNA preparation was used per reaction. Primers against *Pdcd1* and *Actb* (0.4 µM each; online supplemental table 5) and SensiFAST SYBR Hi-ROX reaction mix (Bioline) were added and the reaction was carried out by, and read out by, a StepOnePlus Real-Time PCR system (Thermo Fisher Scientific). Relative gene expression was determined using the ∆∆CT method.^106^

#### Cell-surface protein biotinylation and purification

Cell surface proteins were biotinylated using the EZ-Link Sulfo-NHS-SS-Biotin reagent (Thermo Fisher Scientific) following manufacturer’s instructions. Briefly, cells were washed three times in PBS containing MgCl_2_ and CaCl_2_ and were then resuspended at 1 million per milliliter, and Sulfo-NHS-SS-Biotin was added at a final concentration of 0.1 mg/mL and incubated for 30 min on ice. After incubation, cells were washed three times in quenching buffer (PBS containing MgCl_2_ and CaCl_2_ and 50 mM glycine) before being lysed in IP lysis buffer (30 mM Tris-HCl pH 7.4, 120 mM NaCl, 2 mM EDTA, 2 mM KCl, 1% Triton X-100, supplemented with Halt Protease and Phosphatase Inhibitor), incubated for 30 min and centrifuged at 17,000× g for 10 min. The supernatant, containing protein, was then harvested and labeled protein were immunoprecipitated using Pierce streptavidin beads (Thermo Fisher Scientific) for 1 hour. Proteins were retrieved by boiling samples at 95°C in sample buffer for 5 min. Instead, for mass spectrometry, samples were washed three times in PBS. Western blot was then performed as described above.

#### Immunoprecipitation

48 million CD8 T cells per condition were activated overnight as described above. The next day, cells were harvested, washed twice with PBS and lysed in PD-1-IP lysis buffer (0.5% NP-40 and 2 mM DTT in PBS, pH 7.4) for 30 min. The lysate was then centrifuged at 17,000× g for 10 min. The protein-containing supernatant was harvested, quantified and 8 mg of protein was incubated on a rotator with anti-PD-1 antibody (CST) or isotype control (Thermo Fisher Scientific) for 1 hour at 4°C. After incubation, pre-washed protein A beads (Bio-Rad) were added and incubated for another hour. After IP, the beads were washed three times in lysis buffer and once in PBS, after which bound proteins were retrieved by boiling samples at 95°C in sample buffer for 5 min. Immunoblot analysis of the IPs was performed as per above.

#### RNA sequencing

The total RNA was isolated using the RNeasy Mini Kit (Qiagen), including an on-column DNase digestion according to the manufacturer’s instructions. Quality and quantity of the total RNA were assessed by the 2100 Bioanalyzer using a Nano chip (Agilent). Total RNA samples with RNA Integrity Number (RIN) >8 were subjected to library generation. Strand-specific libraries were generated using the TruSeq Stranded mRNA sample preparation kit (Illumina) according to the manufacturer’s instructions (Illumina). Briefly, polyadenylated RNA from intact total RNA was purified using oligo-dT beads. Following purification, the RNA was fragmented, random primed and reverse transcribed using SuperScript II Reverse Transcriptase (Invitrogen) with the addition of actinomycin D. Second strand synthesis was performed using polymerase I and RNase H with replacement of deoxythymidine triphosphate (dTTP) for deoxyuridine triphosphate (dUTP). The generated cDNA fragments were 3’ end adenylated and ligated to Illumina paired-end sequencing adapters and subsequently amplified by 12 cycles of PCR. The libraries were analyzed on a 2100 Bioanalyzer using a 7500 chip (Agilent, Santa Clara, California, USA), diluted and pooled equimolar into a multiplex sequencing pool. The libraries were sequenced with 65 base single reads on an HiSeq 2500 using V4 chemistry (Illumina).

#### Bioinformatic analyses

Raw read counts were aligned to mouse reference genome GRCm38 Ensembl V.69 using STAR (V.2.7.1a) using two-pass mode and default settings. Differentially expressed genes were identified by DESeq2 (V.1.24). Gene ontology term enrichment of biological process gene sets was performed on significantly differentially expressed genes (false discovery rate (FDR)<0.05) using Panther (V.16.0).^107^ This list was then used as input for REVIGO.^80^ Representative GO terms were then analyzed for directionality by GSEA using GSEA software (V.4.1.0).

For scRNA sequencing analyses, data was downloaded from the TISCH database^108^ (online supplemental table 4). For the correlation between TMED complex expression and T cell dysfunction, we used a 30-gene T cell dysfunction signature computed using MetaCell^18^ (online supplemental table 4). For the analysis of TMED complex expression in exhausted versus other CD8 T cells, populations were defined using canonical markers^109^,^111^ (online supplemental table 4). For each cohort, a mean TMED complex expression across CD8 and exhausted CD8 T cells available in the cohort was computed. The response status of the melanoma cohort was classified according to Response Evaluation Criteria in Solid Tumors (RECIST) for each patient.^12^

For TIL analysis, RNA was extracted from infused TIL products using Tri Reagent (Sigma-Aldrich) according to the manufacturer’s protocol. RNA sequencing libraries were prepared with Illumina’s Ribo Zero Gold and TruSeq stranded library prep kits and sequenced on the Illumina HiSeq 2500 platform using paired-end sequencing with read length of 2×125–150 bps. Reads were aligned to the human genome reference build hg38 using STAR aligner^112^ and were quantified with FeatureCounts.^113^ After filtration of lowly expressed genes (counts below 10 in more than 90% of samples), raw counts were normalized in the R environment according to the LIMMA pipeline.^114^

For RNA sequencing analyses for the ACT experiment, analyses were performed for each isolation condition separately (input, tumor digest and CD8-enriched fractions). Genes with summed counts lower than 10 within one isolation condition were excluded from analysis.

Protein motif searches were performed using MEME.^115^

#### Cytokine release assay

7.5×10^4^ B16F10-OVA cells were seeded in 12-well plate wells and CD8 T cells were added in a 1:8 ratio, in the presence or absence of anti-PD-1 antibody (10 µg/mL; Bio X Cell). After 48 hours, 20 µL supernatant was removed from the well, centrifuged at 1,000× g, and the supernatant was analyzed for the concentration of IFN-γ, TNF and IL-2 by cytometric bead array (BD) following manufacturer’s instructions. For experiments with monocultures, 2 million CD8 T cells were activated in 24-well non-tissue culture-treated plates that were pre-coated with anti-CD3 antibody (for activated cells, 1.25 µg per well) or that were left untreated (for resting cells), instead of activation by tumor cells.

#### Proteomics and analysis

T cell pellets were lysed in heated guanidine lysis buffer as described previously.^116^ Protein concentrations were determined with a Bradford assay (Pierce), after which lysates were diluted to 2 M GuHCl and equal aliquots were taken for a 4-hour trypsin digestion (Sigma-Aldrich, enzyme:protein 1:50) at 37°C, followed by an additional trypsin digestion (1:50) overnight. Digestion was stopped by the addition of 5% formic acid, after which digests were desalted on Sep-Pak C18 cartridges (Waters, Massachusetts, USA). Eluates were dried in a SpeedVac concentrator and stored at −80°C until liquid chromatography tandem mass spectrometry (LC-MS/MS). After reconstitution in 2% formic acid, peptide mixtures were analyzed by nano LC-MS/MS on a Q Exactive HF-X Hybrid Quadrupole-Orbitrap Mass Spectrometer coupled to an EASY-NLC 1,200 system (Thermo Scientific). Samples were loaded directly onto the analytical column (ReproSil-Pur 120 C18-AQ, 1.9 µm, 75 µm×500 mm, packed in-house). Solvent A was 0.1% formic acid/water and solvent B was 0.1% formic acid/80% acetonitrile. For AGN192403-treated and vehicle-treated samples, peptides were eluted from the analytical column at a constant flow of 250 nL/min in a 210 min gradient containing a 195 min linear increase from 6% to 26% solvent B, followed by a 15 min wash. For *Tmed10* KO and WT samples, peptides were eluted with a constant flow of 250 nL/min in a non-linear 210 min gradient containing the following percentages of solvent B: 10% at 5 min; 24% at 130 min; 35% at 170 min; 60% at 190 min and a final wash at 100%. Raw data files were analyzed with label-free quantitation (LFQ) in MaxQuant (V.1.6.17.0)^117^ using standard settings. MS/MS data were searched against the murine Swissprot database (release 2021_04, 17,073 entries) complemented with a list of common contaminants and concatenated with the reversed version of all sequences. The maximum allowed mass tolerance was 4.5 ppm in the main search and 20 ppm for fragment ion masses. FDR for peptide and protein identification were set to 1%. Trypsin/P was chosen as cleavage specificity allowing two missed cleavages. Carbamidomethylation (C) was set as a fixed modification, whereas oxidation (M) and protein N-terminal acetylation were used as variable modifications. LFQ intensities were log2-transformed in Perseus (V.1.6.14.0),^118^ after which protein abundance values were filtered for at least two valid values (out of three total) in at least one condition for the comparison of AGN192403-treated versus vehicle-treated cells, whereas in the case of *Tmed10* KO versus WT cells, protein abundance values were filtered for at least five valid values (out of six total) in at least one condition. Missing values were then replaced by imputation based a normal distribution, using a width of 0.3 and a downshift of 1.8. Differentially regulated proteins were determined using a t-test (thresholds: p<0.05 and a 1.5-fold change in expression).

For the mass spectrometry of cell surface protein-enriched fractions, IP beads were heated for 7 min. at 95°C in 1× S-Trap Lysis buffer (5% SDS, 50 mM TEAB pH 8.5), after which proteins were reduced, alkylated and digested overnight with trypsin (Sigma-Aldrich; 2 µg per sample) on S-Trap Micro spin columns following the manufacturer’s instructions (ProtiFi, New York, USA). Peptides were eluted, vacuum dried and stored at −80°C until LC-MS/MS analysis.

Samples were analyzed by LC-MS/MS on an Exploris 480 mass spectrometer (Thermo Scientific) with a 135 min gradient, the LC set-up being the same as described above. RAW files were analyzed with LFQ in Proteome Discoverer (V.2.5.4.0, Thermo Scientific) using standard settings. MS/MS data were searched against the same database as described above using SEQUEST HT, with the same search parameters and CAMthiopropanoyl (K and N-terminus) as additional variable modifications. The maximum allowed precursor mass tolerance was 50 ppm and 0.06 Da for fragment ion masses, the remaining search parameters being the same as for the T cell proteomes. After filtering for Peptide Spectrum Match (PSM) Xcorr>1, the Proteome Discoverer output file containing protein LFQ abundances was loaded into Perseus and processed as described for the proteome analysis.

#### Human CD8 T cell isolation

Human CD8 T cells were isolated from healthy donor buffycoats as described previously.^102^ Briefly, peripheral blood mononuclear cells (PBMCs) were isolated through Ficoll gradient separation and CD8 T cells were purified by magnetic bead isolation using the Dynabeads CD8 Positive Isolation Kit (Thermo Fisher Scientific) following manufacturer’s instructions. Isolated CD8 T cells were activated for 48 hours on non-tissue culture-treated 24-well plate wells (Corning) that were pre-coated with anti-CD3 (5 µg/well, eBioscience) and anti-CD28 antibodies (5 µg/well, eBioscience). After activation, CD8 T cells were maintained for at least 7 days, refreshing every third day, in T cell medium at a concentration of 1×10^6^ cells/mL before being used for experiments.

#### Murine in vivo ACT experiment

0.5×10^6^ B16F10-OVA cells were injected in both flanks of C57BL/6 mice and allowed to establish for 4 days. After 4 days, mice were randomized and administered PBS (mock), or 2 million sgCtrl or sg*Tmed10* CD8 T cells (which were generated as per above) by intravenous tail injection. Mice received 1E5 units of human IL-2 (Clinigen) two times a day for 3 days after ACT by intraperitoneal injection. Tumor growth was followed by measuring tumor volume three times weekly by calipers, where tumor volume is calculated with the formula: $\frac{\text{length }\left(\text{mm}\right)\text{ × width (mm)}}{\text{2}}$. Seven days after ACT, sentinel tumors were harvested. The rest of the mice were followed until the total tumor volume exceeded 1,500 mm^3^. Mice were given ad libitum access to drinking water, chow and a nutritionally fortified water gel (DietGel).

#### Murine in vivo tumor growth experiment

0.5×10^6^ B16F10-OVA cells were injected in both flanks of C57BL/6 mice and allowed to establish for 4 days. After 4 days, mice were randomized and administered either PBS or AGN192403 (125 mg/kg in PBS) by daily oral gavage for 6 days. Tumor growth was followed by measuring tumor volume three times weekly by calipers, where tumor volume is calculated with the formula: $\frac{\text{length }\left(\text{mm}\right)\text{ × width (mm)}}{\text{2}}$. The day after the final treatment with AGN192403, sentinel tumors were harvested. The rest of the mice were followed until the total tumor volume exceeded 1,500 mm^3^. Mice were given ad libitum access to drinking water, chow and a nutritionally fortified water gel (DietGel).

#### Tumor dissociation and immune cell isolation

Tumors were harvested and cut into ±0.5 mm^2^ pieces and incubated in 5 mL dissociation medium (RPMI with 10 U/mL DNAse I and 200 U/mL collagenase type IV) at 37°C for 1 hour while shaking. After dissociation, tumor preparations were then mashed subsequently through 100 µm and 70 µm cell strainers (Corning) and washed (addition of buffer, followed by centrifugation at 1,000× g for 5 min) in PBS (Gibco) containing 0.1% BSA (Sigma; isolation buffer) before being resuspended and incubated in red blood cell lysis buffer (155 mM NH_4_Cl, 10 mM NaHCO_3_, 0.1 mM EDTA in distilled water; all Sigma) for 5 min. Tumor samples were then washed twice in isolation buffer, before being used in downstream experiments as described above. CD8 cells were isolated from these tumor samples using the Dynabeads FlowComp Mouse CD8 Kit (Thermo Fisher Scientific) following manufacturer’s instructions. RNA sequencing was performed as described above.

#### Quantification and statistical analysis

The details of the quantifications and statistical analyses performed are described in the respective figure legends. Analyses were performed by Prism (GraphPad Software, V.8.4.3) or R. Unless when otherwise specified, a p value of lower than 0.05 was considered statistically significant. Experiments were repeated at least twice, except for the in vivo ACT experiment, which was performed only once. Raw data on which statistics were performed can be found in online supplemental file 2.

## supplementary material

10.1136/jitc-2024-010145 online supplemental figure 1

10.1136/jitc-2024-010145 online supplemental figure 2

10.1136/jitc-2024-010145 online supplemental figure 3

10.1136/jitc-2024-010145 online supplemental figure 4

10.1136/jitc-2024-010145 online supplemental figure 5

10.1136/jitc-2024-010145 online supplemental figure 6

10.1136/jitc-2024-010145 online supplemental figure 7

10.1136/jitc-2024-010145 online supplemental figure 8

10.1136/jitc-2024-010145 online supplemental figure 9

10.1136/jitc-2024-010145 online supplemental table 1

10.1136/jitc-2024-010145 online supplemental table 2

10.1136/jitc-2024-010145 online supplemental table 3

10.1136/jitc-2024-010145 online supplemental table 4

10.1136/jitc-2024-010145 online supplemental table 5

10.1136/jitc-2024-010145 online supplemental file 1

10.1136/jitc-2024-010145 online supplemental file 2

## Data Availability

All data relevant to the study are included in the article or uploaded as supplementary information.
