# Supplementary material for: TMED inhibition suppresses cell surface PD-1 expression and overcomes T cell dysfunction
Source: J Immunother Cancer. 2024 Nov 7;12(11):e010145. doi: 10.1136/jitc-2024-010145 (PMC11552591; doi:10.1136/jitc-2024-010145)
Supplement: online supplemental figure 1 [file jitc-12-11-s001.pdf]

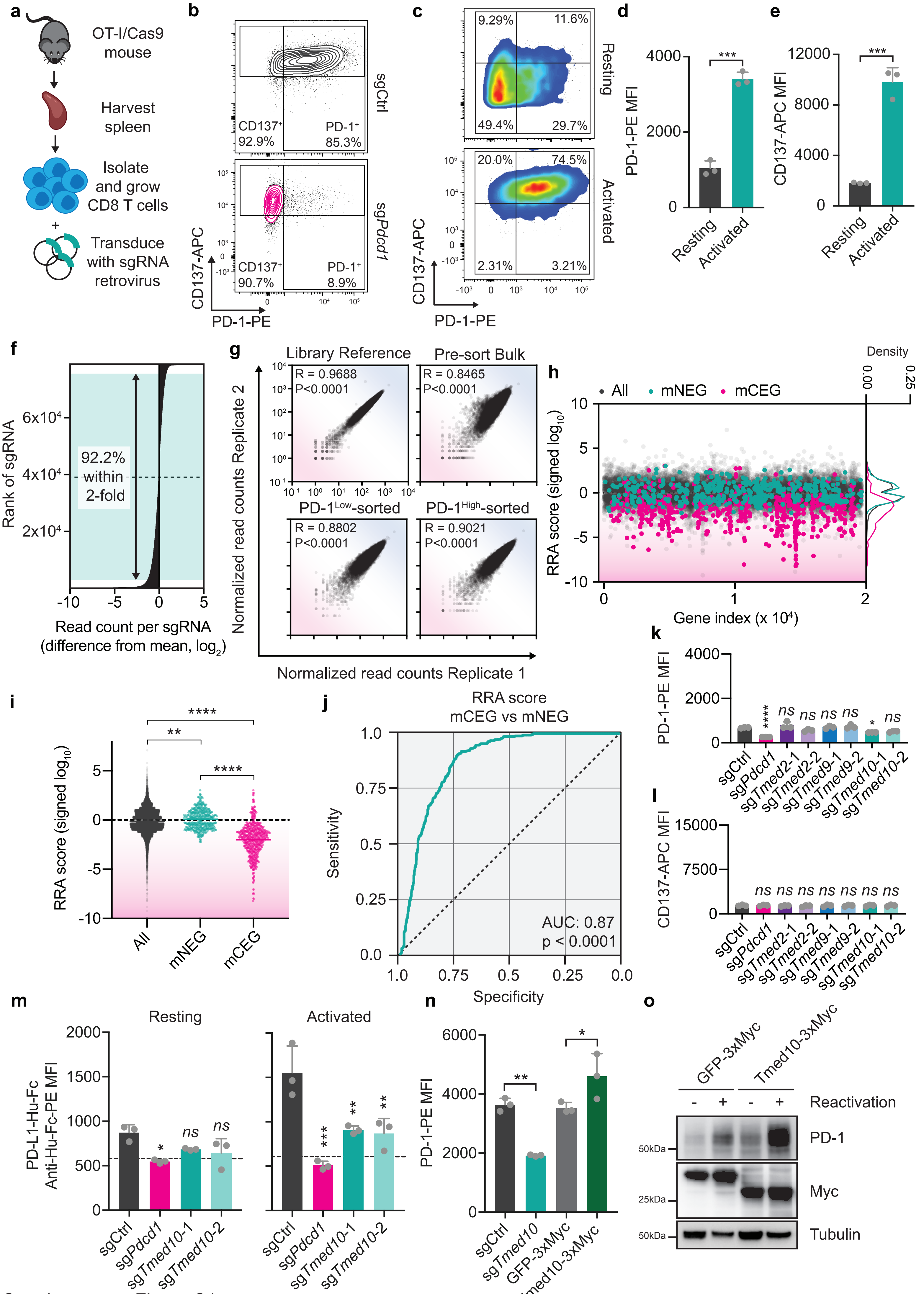

Supplementary Figure S1

Supplementary Figure S1. Quality control analysis of genetic screen and extended validation of regulation of PD-1 abundance by TMED10.

a. Schematic representation of the process by which genetic knockouts were established in primary murine CD8 T cells.

b. Flow cytometry plot of OT-I/Cas9 CD8 T cells harboring a non-targeting control sgRNA, or an sgRNA targeting Pdcd1.

c. Representative flow cytometry plot of resting or activated OT-I/Cas9 CD8 T cells for PD-1-PE (x-axis) and CD137-APC (y-axis).

d. Quantification of experiment in c for PD-1-PE abundance. Each datapoint indicates data obtained with CD8 T cells from an independent spleen. Error bars denote SD. Statistics were performed with a Student t test.

e. As in d, but for CD137-APC abundance.

f. Log2 read counts of sgRNAs in the retroviral Brie library, normalized to the mean read count of the library. Each datapoint indicates single sgRNA, where the sgRNAs are ranked by read count.

g. Pearson correlation plots of normalized read counts of the two replicates for each of the arms of the genetic screen.

h. RRA score (log10) for each of the genes in the PD-1 regulator screen comparing the library reference to the bulk samples. Lower values indicate a depletion of a gene over time. The gene index value refers to the numerical position of a given gene in an alphabetized list of all targeted genes. Statistics were performed by MAGeCK (v0.5.7)63. mNEG, murine non-essential genes; mCEG, murine core essential genes.

i. Quantification of the data in h. Each datapoint indicates a single gene of the indicate group. Statistical analysis was performed by Kruskal-Wallis test with a Dunn’s post hoc test.

j. ROC curve analysis of genes in the mCEG and mNEG groups, using RRA score of h to distinguish these groups.

k. As in Fig. 1f, but for resting cells.

l. As in Fig. 1g, but for resting cells.

m. Labeling of cells with a fusion protein comprising the extracellular domain of PD-L1 and a human antibody Fc domain (PD-L1-Fc) followed by quantification of Fc-PE abundance by flow cytometry for resting (left) or activated (right) OT-I/Cas9 CD8 T cells carrying indicated sgRNAs. Each datapoint indicates data obtained with CD8 T cells from an independent spleen. Error bars denote SD. Statistical analysis was performed with a one-way ANOVA, followed by Tukey post hoc analysis. The dotted line indicates the MFI obtained with sgCtrl cells that were not labeled with PD-L1-Fc.

n. Quantification of PD-1-PE abundance by flow cytometry for activated OT-I/Cas9 CD8 T cells carrying indicated constructs. Each datapoint indicates data obtained with CD8 T cells from an independent spleen. Error bars denote SD. Statistical analysis was performed with a one-way ANOVA, followed by Tukey post-hoc analysis.

o. Western blot analysis of PD-1 and Myc abundance in OT-I/Cas9 CD8 T cells carrying the indicated constructs before or after activation with anti-CD3 antibody for 24h. The size markings indicate the size of the closest molecular weight marker.

* $P < 0.05$; ** $P < 0.01$; *** $P < 0.001$; **** $P < 0.0001$.
