# Supplementary material for: TMED inhibition suppresses cell surface PD-1 expression and overcomes T cell dysfunction
Source: J Immunother Cancer. 2024 Nov 7;12(11):e010145. doi: 10.1136/jitc-2024-010145 (PMC11552591; doi:10.1136/jitc-2024-010145)
Supplement: online supplemental figure 2 [file jitc-12-11-s002.pdf]

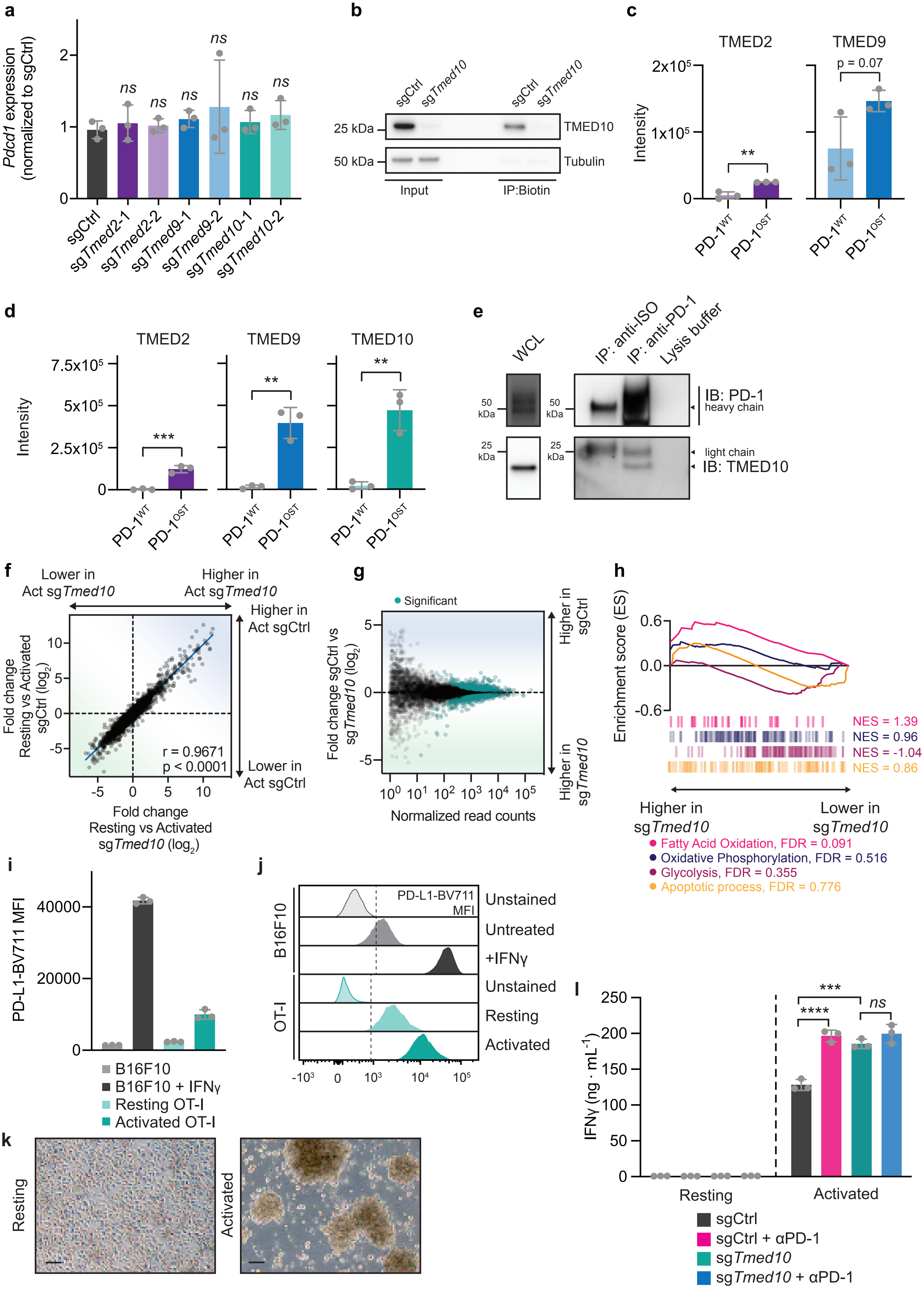

Supplementary Figure S2

Supplementary Figure S2. Extended analyses of the biological consequences of Tmed10 inactivation in CD8 T cells.

a. Quantification of Pdcd1 RNA expression in activated OT-I/Cas9 CD8 T cells by qPCR carrying the indicated sgRNAs. Expression data was normalized to the internal reference Actb and then normalized again to the expression of Pdcd1 in the sgCtrl sample following the ΔΔCt method103. Each datapoint indicates data obtained with CD8 T cells from an independent spleen. Error bars denote SD. Statistical analysis was performed by one-way ANOVA, followed by a Dunnett post-hoc test.

b. Western blot analysis of TMED10 in activated OT-I/Cas9 CD8 T cells carrying the indicated sgRNA constructs labeled with an amine-reactive, non-membrane permeable biotinylation reagent before (input) or after (IP) immunoprecipitation with streptavidin beads. The size markings indicate the size of the closest molecular weight marker.

c. As in Fig. 2a, but for multiple TMED2 and TMED9 proteins.

d. As in Fig. 2a and c, but for human Jurkat T cells.

e. Western blot analysis of PD-1 and TMED10 in activated OT-I/Cas9 CD8 T cells before (WCL) or after (co-)immunoprecipitation with an isotype control (IP: anti-ISO) or PD-1 (IP: anti-PD-1) antibody. The size markings indicate the size of the closest molecular weight marker.

f. Pearson correlation plot of transcriptomic changes between resting and activated OT-I/Cas9 CD8 T cells carrying a non-targeting control sgRNA (y-axis) and resting and activated OT-I/Cas9 CD8 T cells carrying an sgRNA targeting Tmed10 (x-axis). Each datapoint indicates the relative changes in expression of a single gene.

g. Differential transcriptome analysis plot, comparing OT-I/Cas9 CD8 T cells carrying a non-targeting control sgRNA and cells carrying an sgRNA targeting Tmed10. Statistics were performed by a Wald test, with genes being called differentially expressed when FDR < 0.1.

h. As in Fig. 2D, but for the gene sets REACTOME_MITOCHONDRIAL_FATTY_ACID_BETA_OXIDATION, GO_OXIDATIVE_PHOSPHORYLATION_TMED, REACTOME_GLYCOLYSIS and GO_ LEUKOCYTE_APOPTOTIC_PROCESS.

i. Quantification of indicated samples for PD-L1-BV711 abundance by flow cytometry. Each datapoint indicates data obtained with CD8 T cells from an independent spleen or, in the case of the B16F10-OVA cells, three independent cultures. B16F10-OVA cells were treated, or not, with 25ng/mL IFNγ for 24hrs to induce PD-L1. OT-I CD8 T cells were activated using anti-CD3-antibodies for 24hrs. Error bars denote SD.

j. Representative flow cytometry histograms of PD-L1-BV711 of the samples in i.

k. Representative microscopic images of resting and activated OT-I CD8 T cells from i. The scale bar indicates a length of 50μm.

l. Cytokine release of IFNγ as measured by cytometric bead array of sgCtrl or sgTmed10 OT-I/Cas9 CD8 T cells, after activation, or not, with anti-CD3-antibodies in the presence or absence of anti-PD-1 antibody (10 μg/mL). Each datapoint indicates data obtained with CD8 T cells from an independent spleen. Error bars denote SD. Statistical analysis was performed with a one-way ANOVA, followed by a Tukey post-hoc test.

* P < 0.05; ** P < 0.01; *** P < 0.001; **** P < 0.0001.
