# Supplementary material for: TMED inhibition suppresses cell surface PD-1 expression and overcomes T cell dysfunction
Source: J Immunother Cancer. 2024 Nov 7;12(11):e010145. doi: 10.1136/jitc-2024-010145 (PMC11552591; doi:10.1136/jitc-2024-010145)
Supplement: online supplemental figure 3 [file jitc-12-11-s003.pdf]

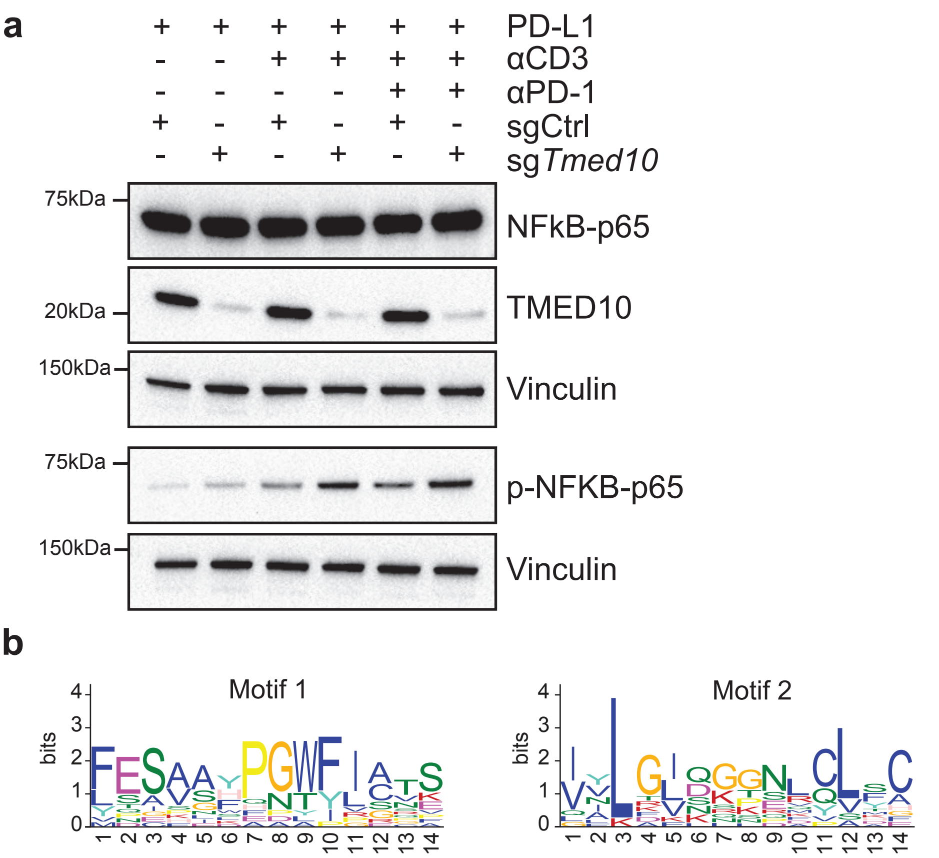

c

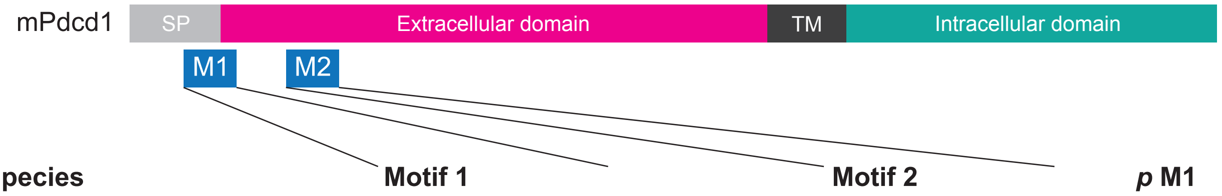

Species		Motif 1		Motif 2		*p* M1	*p* M2
Bos taurus	WAV	LQLGCWPGWLLEAS	SRPWSALTFSPP	RLVVPEGANATFTC	SFS	$9.6x10^{-4}$	8.3x10-$^{-3}$
Mesocricetus auratus	WAV	LQFSWQAGWLLEVS	SGSWRPLTFSPA	WLTVSEGSNATFTC	SFS	$7.9x10^{-3}$	8.3x10-$^{-3}$
Mus musculus	WAV	LQLSWQSGWLLEVP	NGPWRSLTFYPA	WLTVSEGANATFTC	SLS	$4.3x10^{-2}$	8.3x10-$^{-3}$
Rattus norvegicus	WAV	LQLSWQSGWLLEVL	NKPWRPLTFSPT	WLTVSEGANATFTC	SFS	$4.3x10^{-2}$	$8.3x10^{-3}$
Homo sapiens	WAV	LQLGWRPGWFLDSP	DRPWNPPTFSPA	LLVVTEGDNATFTC	SFS	$7.5x10^{-4}$	$8.3x10^{-3}$
Gorilla gorilla	WAV	LQLGWRPGWFLDSP	DRPWNPPTFSPA	LLVVTEGDNATFTC	SFS	$7.5x10^{-4}$	$8.3x10^{-3}$
Canis lupus	WAV	LQLGWWPGWLLDSP	DRPWSPLTFSPA	QLTVQEGENATFTC	SLA	$6.9x10^{-3}$	$8.3x10^{-3}$
Felis catus	WAV	LQLGWWPGWLLDSP	YRPWSPLTFSPA	QLTVLEGENATFVC	HLP	$6.9x10^{-3}$	$8.3x10^{-3}$
Ornithorhynchus anatinus	FYT	VLLGCQPRLLRAQI	KSEASCSASSPA	KLFGQKGENFTFFC	SVS	$4.9x10^{-3}$	$1.2x10^{-3}$

d

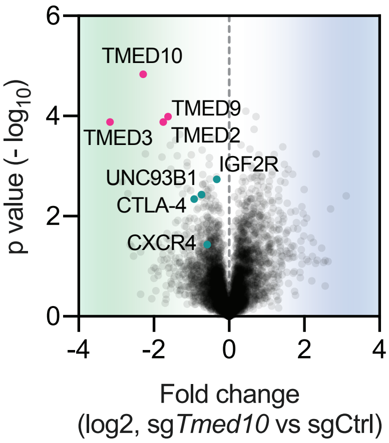

e

Name	Motif	Start	p-value
CTLA-4	2	112	2.0 x 10^{-6}
SGTFNESR	VNLTIQGLRAVDTG	LYLCKVEL	
**Name**	**Motif**	**Start**	**p-value**
UNC93B1	2	77	9.4 x 10^{-5}
TGVTLTYG	VYLGLLQMQLILHY	DETYREVK	
**Name**	**Motif**	**Start**	**p-value**
CXCR4	2	127	5.9 x 10^{-5}
TVNLYSSV	LILAFISLDRYLAI	VHATNSQR	
**Name**	**Motif**	**Start**	**p-value**
IGF2R	2	1253	9.1 x 10^{-5}
VGEYTYYL	RVCGKLSSDVCSAH	GSKAVSSC	

f

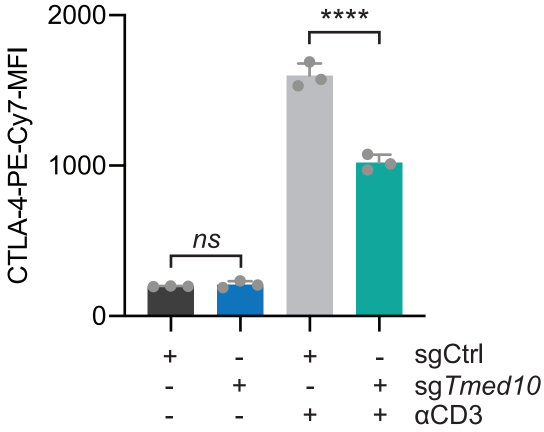

Supplementary Figure S3

Supplementary Figure S3. TMED10 co-regulates other client proteins including CTLA-4.

a. Western blot analysis of indicated proteins in resting or activated OT-I/Cas9 CD8 T cells carrying the indicated sgRNA constructs, in the presence of recombinant PD-L1 and, in some conditions, anti-PD-1 (10µg/mL) after 1 hour of treatment. The size markings indicate the size of the closest molecular weight marker.

b. Sequence logos of putative TMED10 protein regulation motifs72.

c. Motif scanning alignment plots of the motifs in b in the protein sequence of PD-1 in multiple, indicated species. P values were calculated by MEME104.

d. Proteomic differences in cell-surface protein fractions between OT-I/Cas9 CD8 T cells carrying sgCtrl or sgTmed10 after activation with CD3 antibody for 24h as measured by mass spectrometry. The data is based on three independent spleens for each treatment. Statistical analysis was performed by a Student's t test. Proteins highlighted in teal are the only proteins that carry TMED10 protein regulation motif as well as being differentially present in the cell-surface fraction between the two genotypes.

e. Motif scanning alignment plots of the motifs in b in the protein sequences of the highlighted proteins in d. P values were calculated by MEME.

f. Quantification of flow cytometry measurements for the abundance of CTLA-4-PE-Cy7 in OT-I/Cas9 CD8 T cells of the indicated genotypes and activation status. Each datapoint indicates data obtained with CD8 T cells from an independent spleen. Error bars denote SD. Statistics were performed with a Student t test for each activation condition.

* P < 0.05; ** P < 0.01; *** P < 0.001; **** P < 0.0001.
