# Supplementary material for: TMED inhibition suppresses cell surface PD-1 expression and overcomes T cell dysfunction
Source: J Immunother Cancer. 2024 Nov 7;12(11):e010145. doi: 10.1136/jitc-2024-010145 (PMC11552591; doi:10.1136/jitc-2024-010145)
Supplement: online supplemental figure 4 [file jitc-12-11-s004.pdf]

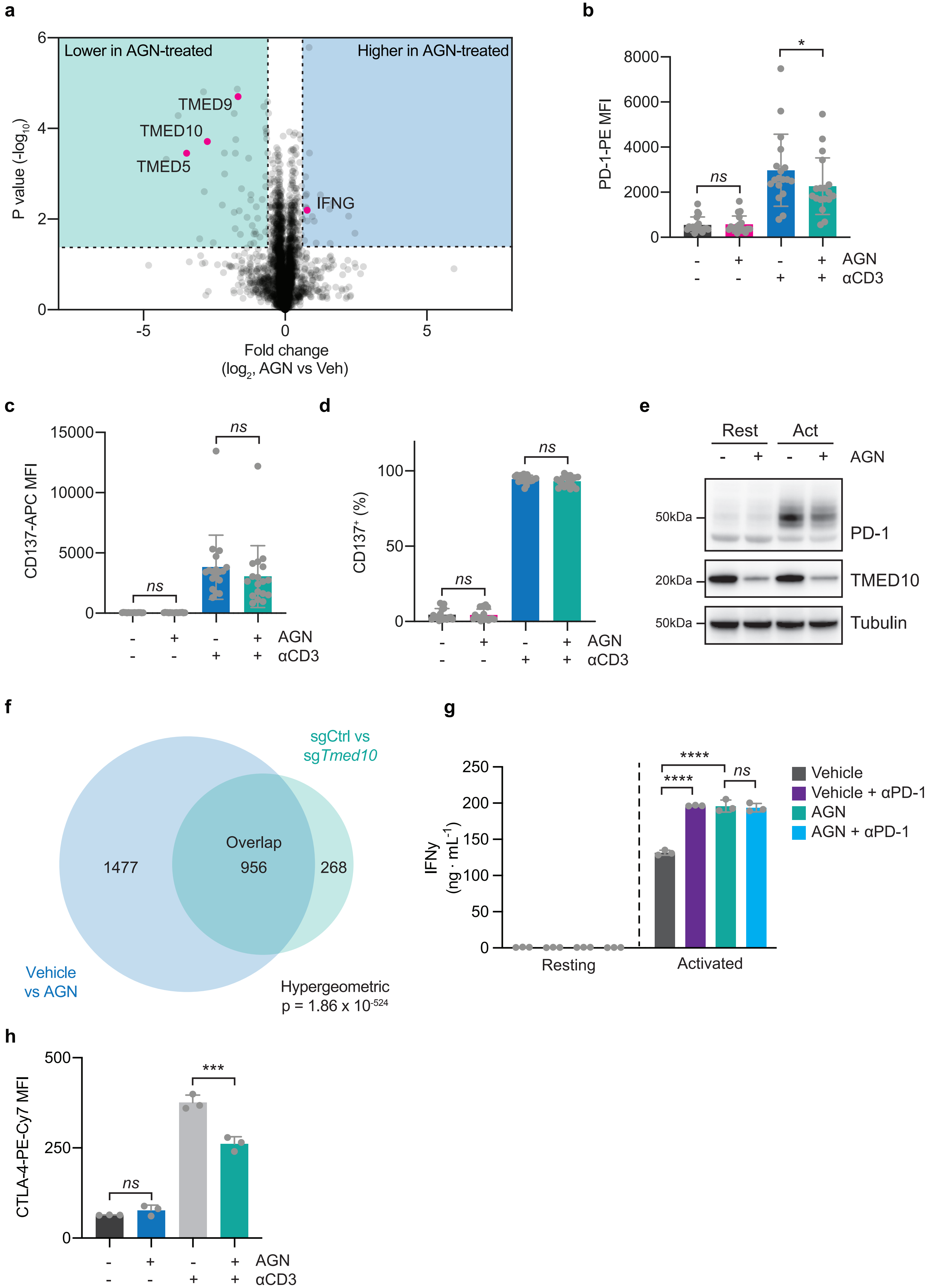

Supplementary Figure S4

Supplementary Figure S4. AGN192403 phenocopies Tmed10 KO in murine and human CD8 T cells.

a. Proteomic differences between OT-I/Cas9 CD8 T cells treated with vehicle or AGN192403 (AGN, 100µM) after activation with anti-CD3 antibody for 24h as measured by mass spectrometry. The data is based on three independent spleens for each treatment. Statistical analysis was performed by a Student's t test.

b. Quantification of PD-1-PE abundance by flow cytometry of resting and activated human CD8 T cells in the presence or absence of AGN192403 (AGN; 100µM). Each datapoint indicates data obtained with CD8 T cells from independent donors. Error bars denote SD. Statistical analysis was performed with a Friedman test, followed by a Dunn's post-hoc analysis.

c. As in b, but for CD137-APC abundance.

d. As in b, but for percentage of CD137+ cells.

e. Western blot analysis of PD-1 and TMED10 abundance in OT-I/Cas9 CD8 T cells, before or after activation with anti-CD3 antibody for 24h in the presence or absence of AGN192403 (AGN; 100 µM). The size markings indicate the size of the closest molecular weight marker.

f. Overlap of significantly differentially expressed genes between activated OT-I/Cas9 CD8 T cells carrying either a non-targeting control sgRNA or an sgRNA targeting Tmed10 (green) and activated OT-I/Cas9 CD8 T cells treated with vehicle or AGN192403 (AGN; 100µM; blue). Statistical p-value was determined by hypergeometric test.

g. Cytokine release of IFNγ as measured by cytometric bead array of vehicle- or AGN192403-treated OT-I/Cas9 CD8 T cells, after activation, or not, with CD3-antibody in the presence or absence of PD-1 antibody (10 µg/mL). Each datapoint indicates data obtained with CD8 T cells from an independent spleen. Error bars denote SD. Statistical analysis was performed with a one-way ANOVA, followed by a Tukey post-hoc test.

h. Quantification of flow cytometry measurements for the abundance of CTLA-4-PE-Cy7 in resting and activated OT-I/-Cas9 CD8 T cells treated with, or not, AGN192403. Each datapoint indicates data obtained with CD8 T cells from an independent spleen. Error bars denote SD. Statistics were performed with a Student t test for each activation condition.

* P < 0.05; ** P < 0.01; *** P < 0.001; **** P < 0.0001.
