# Supplementary material for: TMED inhibition suppresses cell surface PD-1 expression and overcomes T cell dysfunction
Source: J Immunother Cancer. 2024 Nov 7;12(11):e010145. doi: 10.1136/jitc-2024-010145 (PMC11552591; doi:10.1136/jitc-2024-010145)
Supplement: online supplemental figure 5 [file jitc-12-11-s005.pdf]

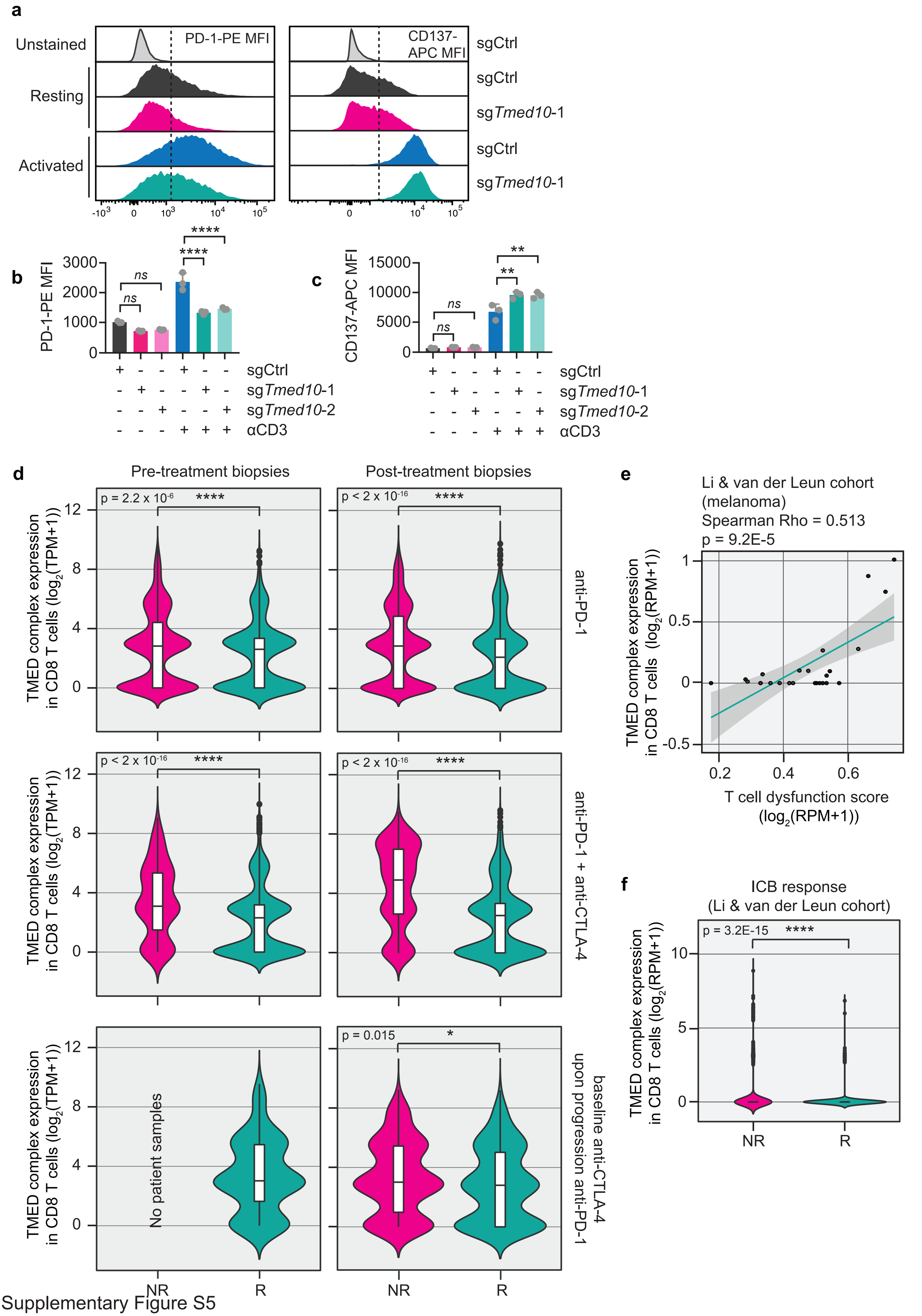


Supplementary Figure S5

Supplementary Figure S5. Detailed analysis of TMED complex abundance in human and mouse CD8 T cells.

a. As Fig. 4a, but for PD-1-PE abundance (left) and CD137-APC abundance (right).
b. As Fig. 4b, but for PD-1-PE abundance.
c. As b, but for CD137-APC abundance.
d. As Fig. 4e, but the cohort was split up based on time of biopsy and treatment regimen.
e. As in Fig. 4c, but for the Li & van der Leun cohort18.
f. As in Fig. 4e, but for the Li & van der Leun cohort18.

* P < 0.05; ** P < 0.01; *** P < 0.001; **** P < 0.0001.

.
