# Supplementary material for: TMED inhibition suppresses cell surface PD-1 expression and overcomes T cell dysfunction
Source: J Immunother Cancer. 2024 Nov 7;12(11):e010145. doi: 10.1136/jitc-2024-010145 (PMC11552591; doi:10.1136/jitc-2024-010145)
Supplement: online supplemental figure 6 [file jitc-12-11-s006.pdf]

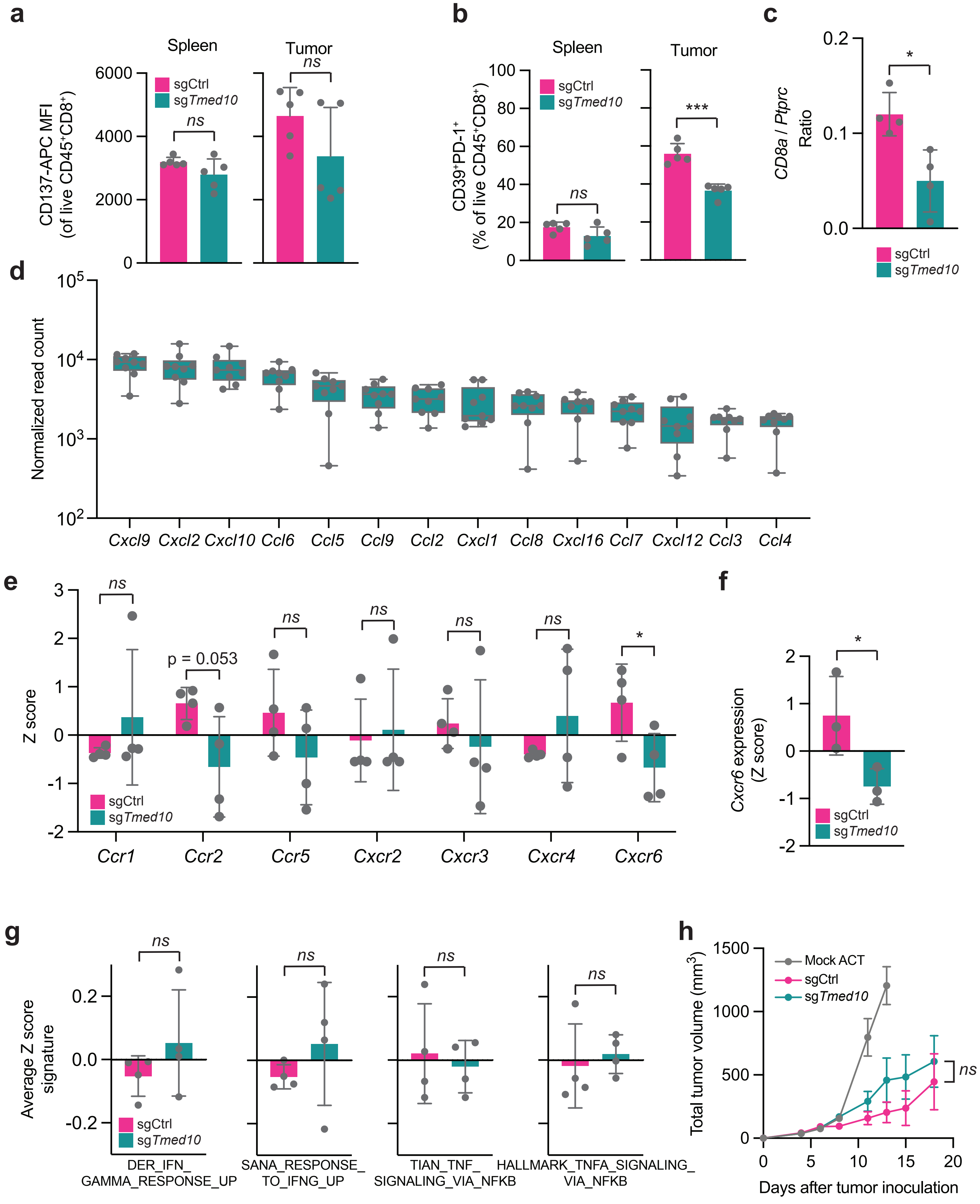

Supplementary Figure S6

Supplementary Figure S6. Lack of anti-tumor effect of ACT with sgTmed10 CD8 T cells in murine melanoma.

a. As in Fig. 5b, but for CD137-APC MFI.

b. Quantification of CD39+PD-1+ cells within the live, CD45+CD8+ cell population in the indicated tissues. Each datapoint indicates data obtained with an independent tumor. Error bars denote SD. Statistical analysis was performed with a Student t test.

c. Ratio of normalized read counts of CD8a and Ptprc genes in tumors of mice that received ACT with CD8 T cells carrying sgCtrl or sgTmed10. Each datapoint indicates data obtained with an independent tumor. Error bars denote SD. Statistical analysis was performed with a Student t test. Each datapoint indicates data obtained with an independent tumor.

d. Normalized read counts of expressed chemokines in B16F10 tumors as measured by RNA sequencing. Each datapoint indicates data obtained with an independent tumor, with data of tumors of mice that received ACT with CD8 T cells carrying sgCtrl or sgTmed10 visualized in the same plot. In the boxplots, the center line, box edges and whiskers denote the median, interquartile range and the rest of the distribution respectively, with outliers being shown separately.

e. Z-score expression of the indicated genes in CD8-enriched tumor fractions of mice that received ACT with CD8 T cells carrying sgCtrl or sgTmed10. Each datapoint indicates the expression of the indicated gene in the CD8+ TIL population of an individual mouse. Error bars denote SD. Statistical analysis was performed with a Student t test for each chemokine receptor.

f. Z-score expression of Cxcr6 in CD8 T cells carrying sgCtrl or sgTmed10 before use in ACT. Each datapoint indicates the expression of Cxcr6 in one of the technical replicates analyzed by RNA sequencing. Error bars denote SD. Statistical analysis was performed with a Student t test for each chemokine receptor.

a. Average Z scores of indicated immune response signatures in B16F10 tumors as measured by RNA sequencing. Each datapoint indicates data obtained with an independent tumor, with data of tumors of mice that received ACT with CD8 T cells carrying sgCtrl or sgTmed10 visualized in separate plots. In the boxplots, the center line, box edges and whiskers denote the median, interquartile range and the rest of the distribution respectively, with outliers being shown separately. Error bars denote SD. Statistical analysis was performed with a Student t test for each signature. Tumor growth curves of the experiment in Fig. 5a. Each datapoint indicates the average tumor volume of each cohort of mice. Error bars denote SEM. The curves were stopped when the first mouse had to be sacrificed due to maximum tumor volume. Statistics were performed by a Student t test at each timepoint.

* P < 0.05; ** P < 0.01; *** P < 0.001; **** P < 0.0001.
