# Supplementary material for: TMED inhibition suppresses cell surface PD-1 expression and overcomes T cell dysfunction
Source: J Immunother Cancer. 2024 Nov 7;12(11):e010145. doi: 10.1136/jitc-2024-010145 (PMC11552591; doi:10.1136/jitc-2024-010145)
Supplement: online supplemental figure 7 [file jitc-12-11-s007.pdf]

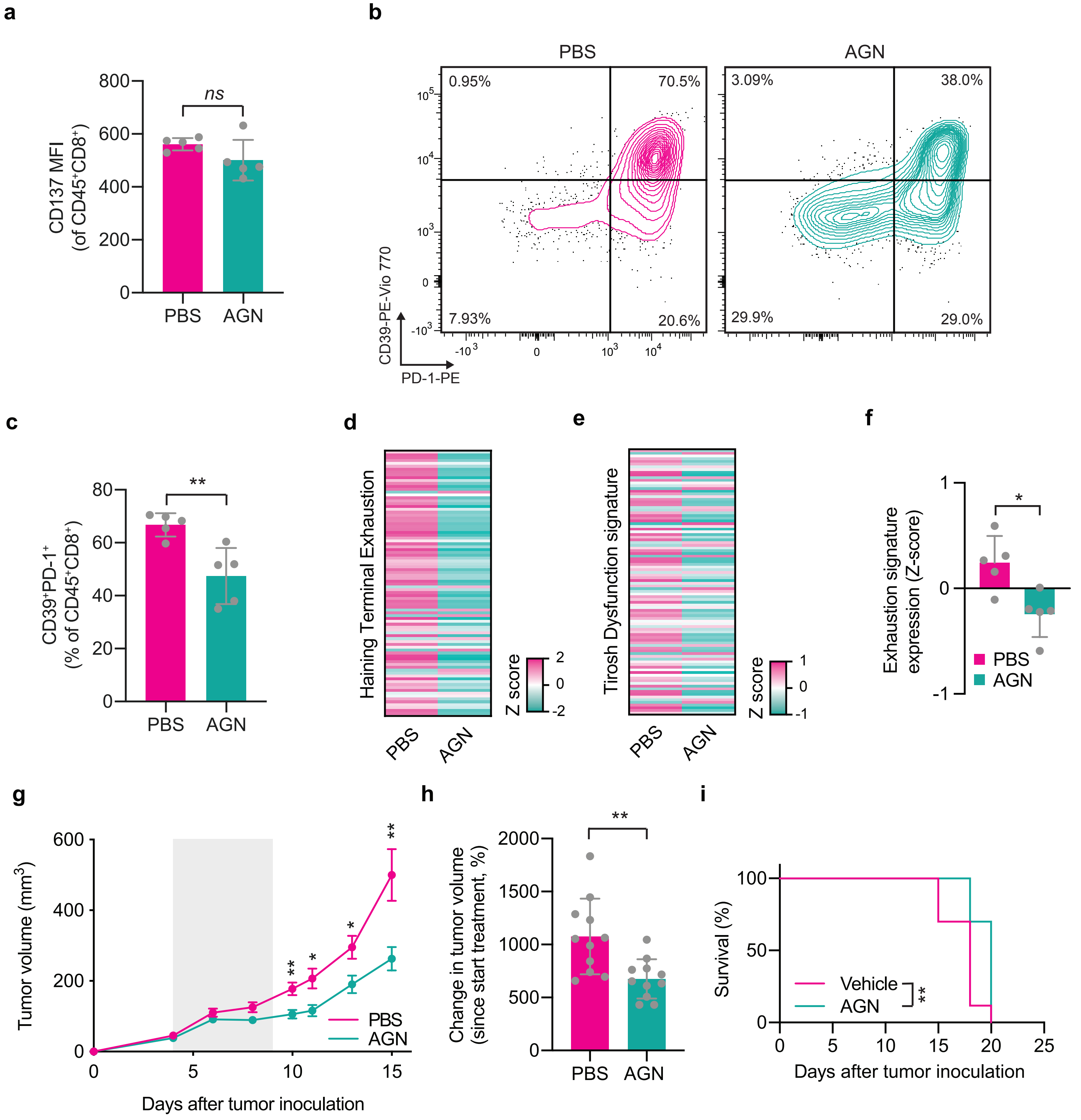

Supplementary Figure S7

Supplementary Figure S7. Anti-tumor effect of AGN192403 therapy in murine melanoma.

b. As in Figure 5h, but for CD137-APC abundance.

c. Representative flow cytometry plots for PD-1-PE abundance (x-axis) and CD39-PE-Vio770 abundance (y-axis) of tumor-infiltrating, live, CD45+CD8+ cells in mice treated with either PBS (left) or AGN192403 (AGN; right).

d. Quantification of experiment in b. Each datapoint indicates data obtained with an independent tumor. Error bars denote SD. Statistical analysis was performed with a Student t test.

e. Z-score expression of individual genes in the Terminal Exhaustion gene set from Figure 5i. Each datapoint indicates the average expression of the five CD8+ TIL samples analyzed per condition.

f. As in d, but for the Tirosh Dysfunction signature.

g. As in Figure 5j, but for the Tirosh Dysfunction signature.

h. Tumor growth curves of the experiment in Fig. 5e. The shaded area indicates period of treatment with AGN192403 (AGN; teal) or vehicle (pink) by daily oral gavage. Each datapoint indicates the average tumor volume of each cohort of mice. Error bars denote SEM. The curves were stopped when the first mouse had to be sacrificed due to maximum tumor volume. Statistics were performed by a Student t test at each timepoint.

i. Percentage tumor growth since day 4, until day 15. Each tumor volume was normalized to its own size at day 4, the start of treatment. Each datapoint indicates data obtained from a single mouse. Statistics were performed by a Student t test.

j. Kaplan-Meier survival curve of experiment in h. Statistical testing was performed by Log-rank test.
