# Supplementary material for: TMED inhibition suppresses cell surface PD-1 expression and overcomes T cell dysfunction
Source: J Immunother Cancer. 2024 Nov 7;12(11):e010145. doi: 10.1136/jitc-2024-010145 (PMC11552591; doi:10.1136/jitc-2024-010145)
Supplement: online supplemental figure 8 [file jitc-12-11-s008.pdf]

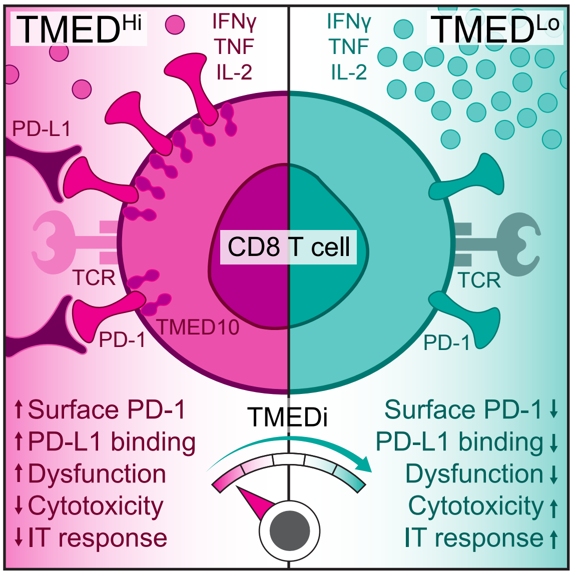

Supplementary Figure S8. TMED10 controls T cell (dys)function by regulating cell-surface PD-1 abundance. TCR, T cell receptor; IT, immunotherapy.
