# Supplementary material for: TMED inhibition suppresses cell surface PD-1 expression and overcomes T cell dysfunction
Source: J Immunother Cancer. 2024 Nov 7;12(11):e010145. doi: 10.1136/jitc-2024-010145 (PMC11552591; doi:10.1136/jitc-2024-010145)
Supplement: online supplemental figure 9 [file jitc-12-11-s009.pdf]

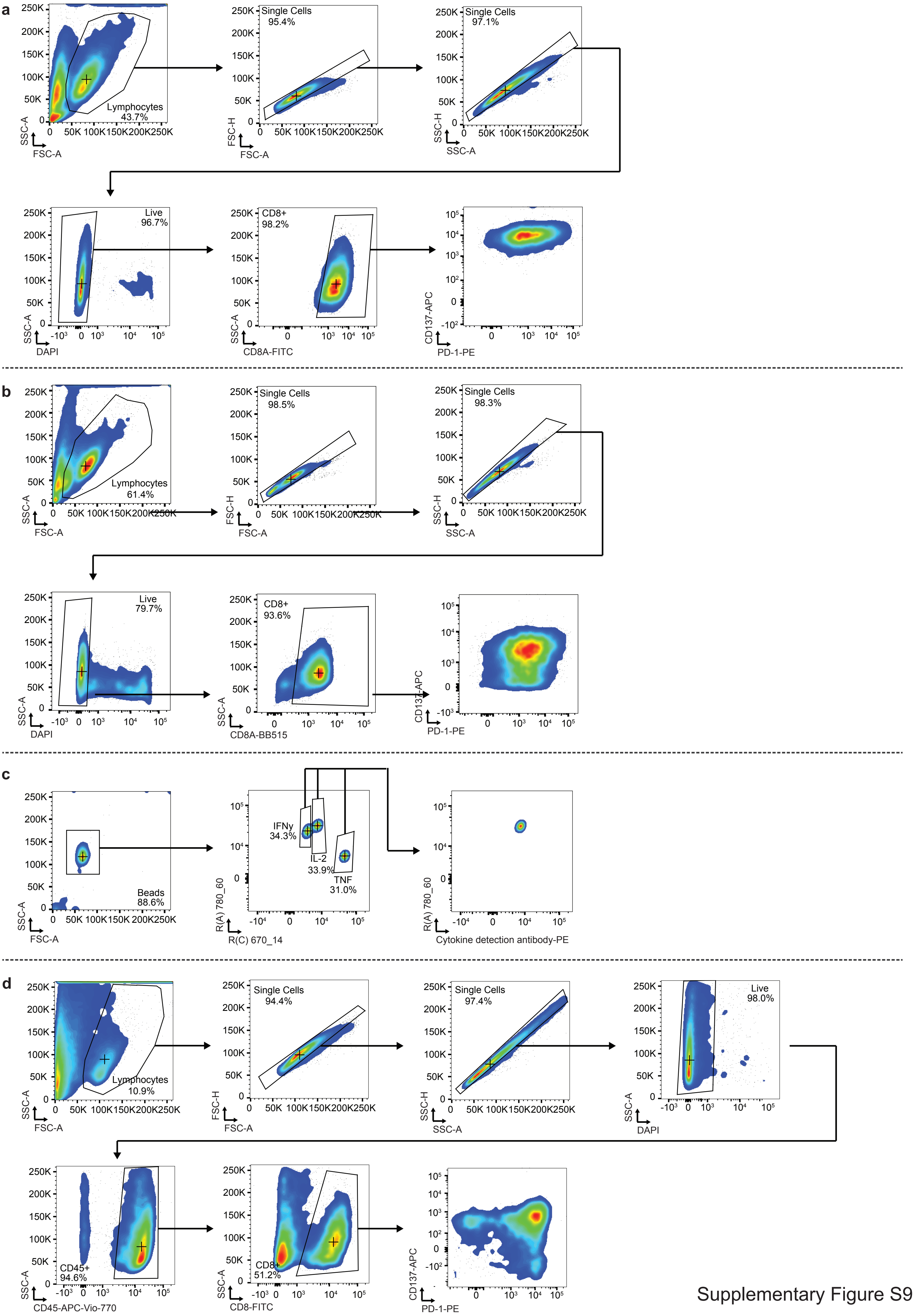

Supplementary Figure S9

Supplementary Figure S9. Gating strategy for FACS experiments.

a. Gating strategy corresponding to Figures 1e-g, 2b, 2c, 3b-e, 4a, b and Extended Data Figures 1b-e, k-o, 3f, 4h and 5a-c.

b. Gating strategy corresponding to Extended Data Figures 4b-d.

c. Gating strategy corresponding to Figures 2g, 3j and Extended Data Figures 2l and 4g.

d. Gating strategy corresponding to Figures 5b-d, 5h and Extended Data Figures 6a, 6b, and 7a-c.
