# Supplementary material for: TMED inhibition suppresses cell surface PD-1 expression and overcomes T cell dysfunction
Source: J Immunother Cancer. 2024 Nov 7;12(11):e010145. doi: 10.1136/jitc-2024-010145 (PMC11552591; doi:10.1136/jitc-2024-010145)
Supplement: online supplemental table 5 [file jitc-12-11-s014.pdf]

Identifier	Sequence (5’-3’)
Sequencing primer, Brie_Fw	AATGATACGGCGACCACCGAGATCTACACTCTTTCCCTACACGACGCTCTTCCGATCTNNNNNNGGCTTTATATATCTTGTGGAAAGGACGAAACACC
Sequencing primer, Brie_Rv	CAAGCAGAAGACGGCATACGAGATCCGACTCGGTGCCATTTTTCAA
Non-targeting control sgRNA	GTATTACTGATATTGGTGGG
Pdcd1 sgRNA	GCTCAAACCATTACAGAAGG
Tmed2 sgRNA 1	CCGCTCGAAGAAGCACTCCT
Tmed2 sgRNA 2	GGACAAGACATGGAGACAGA
Tmed9 sgRNA 1	GAAATACAGCGCGCTTCCGC
Tmed9 sgRNA 2	GTCATCGGTGCGGGGGCGCA
Tmed10 sgRNA 1	CTCTCGCAAGTGTCTCCGAG
Tmed10 sgRNA 2	AAACATGTCATAGTCTTCCG
qPCR primer, *Pdcd1*-Fw	TGGTCATTCACTTGGGCTGT
qPCR primer, *Pdcd1*-Rv	TGGGTAGAAGGTGAGGGACC
qPCR primer, *Actb1*-Fw	GATCTGGCACCACACCTTCT
qPCR primer, *Actb1*-Rv	GGGGTGTTGAAGGTCTCAAA
