# Supplementary figures and images for: TMED inhibition suppresses cell surface PD-1 expression and overcomes T cell dysfunction
Source: J Immunother Cancer. 2024 Nov 7;12(11):e010145. doi: 10.1136/jitc-2024-010145 (PMC11552591; doi:10.1136/jitc-2024-010145)

Figure 1H

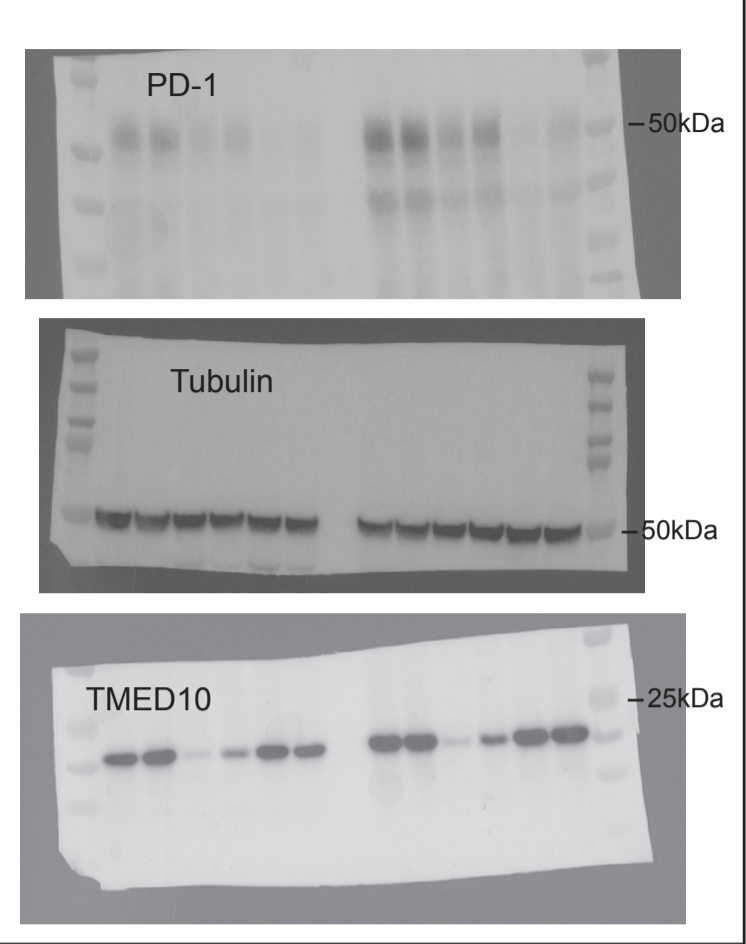

Figure 2I

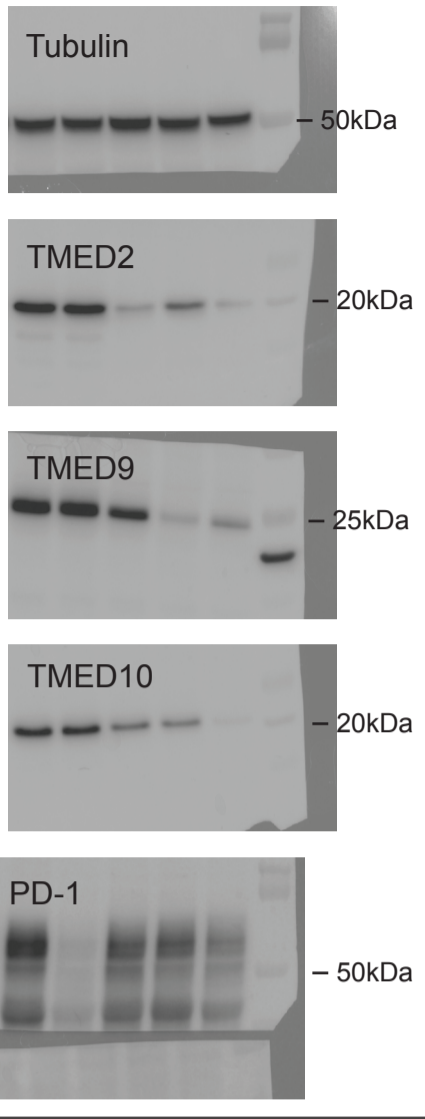

Figure 3A

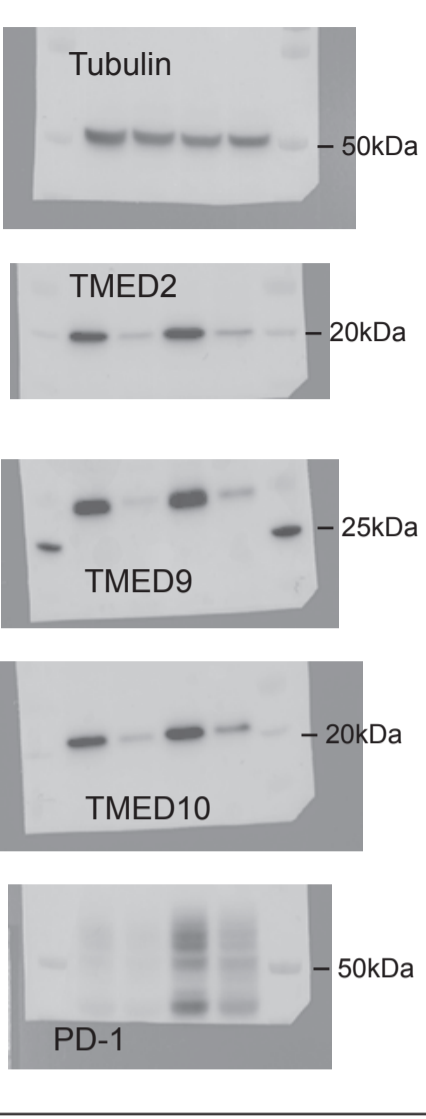

Figure 3F

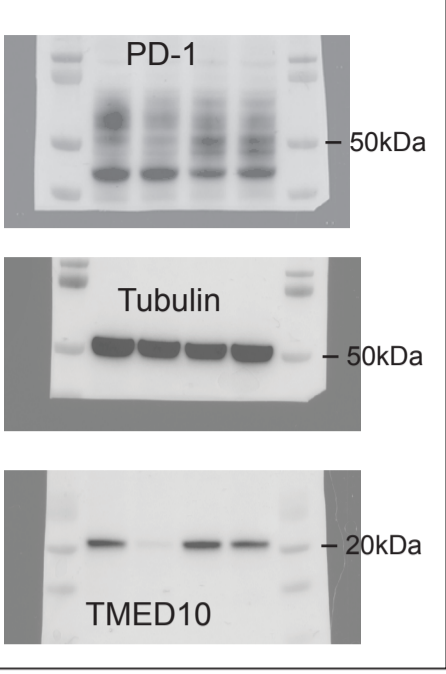

Figure 5F

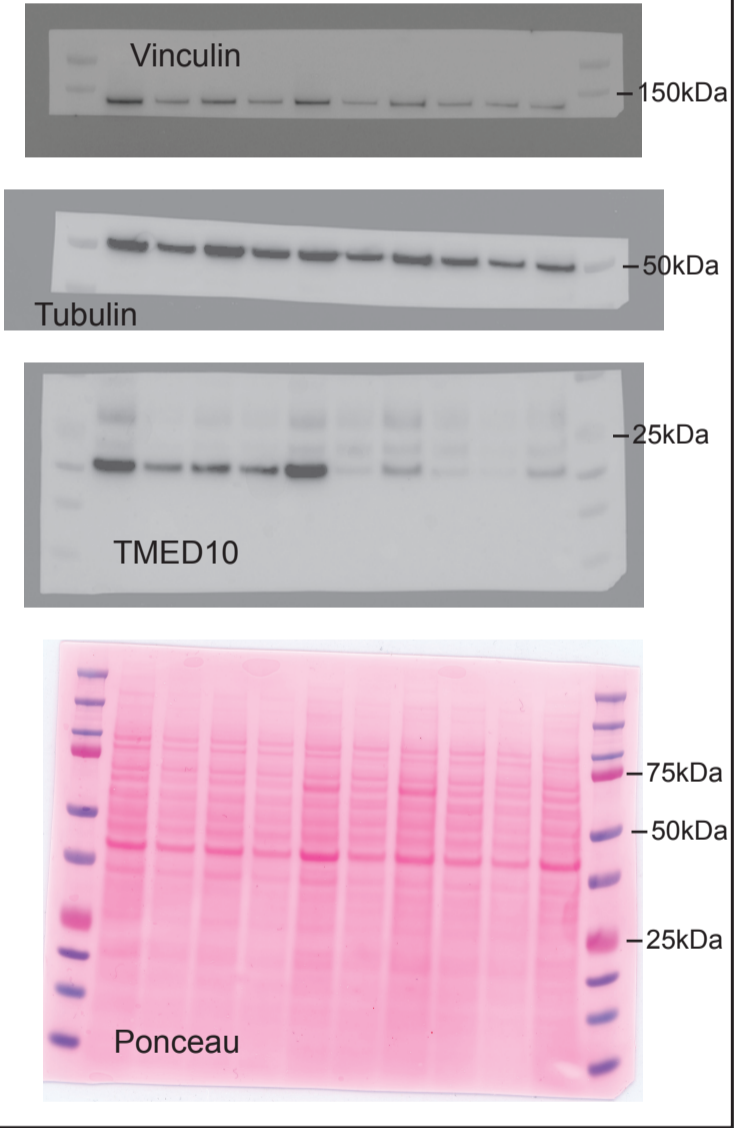

Figure S1O

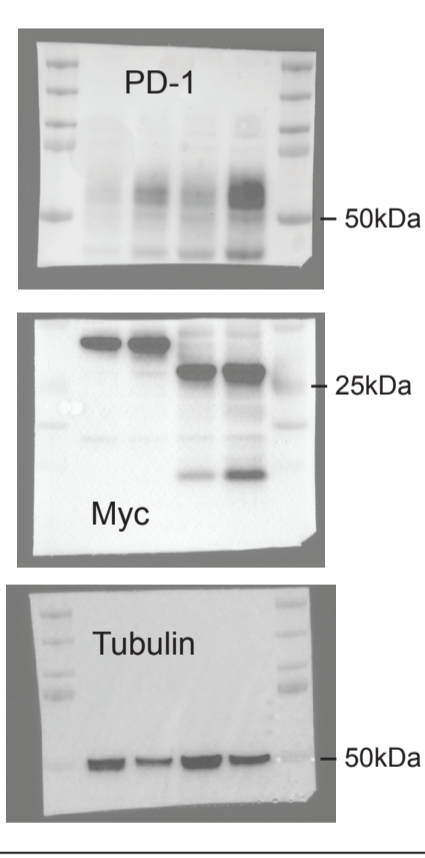

Figure S2B

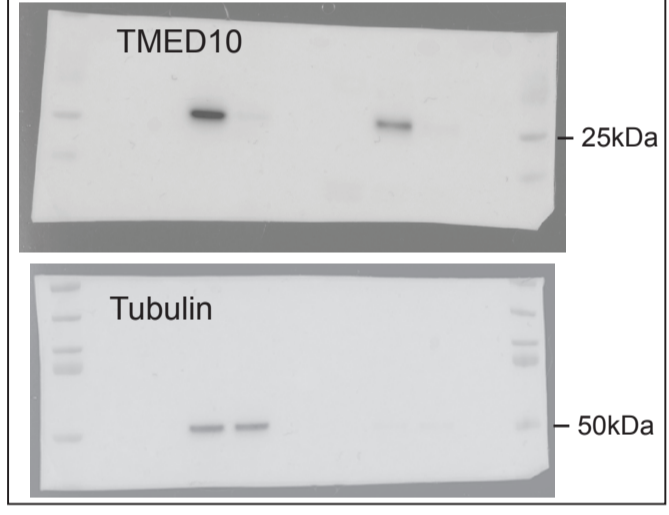

Figure S2E

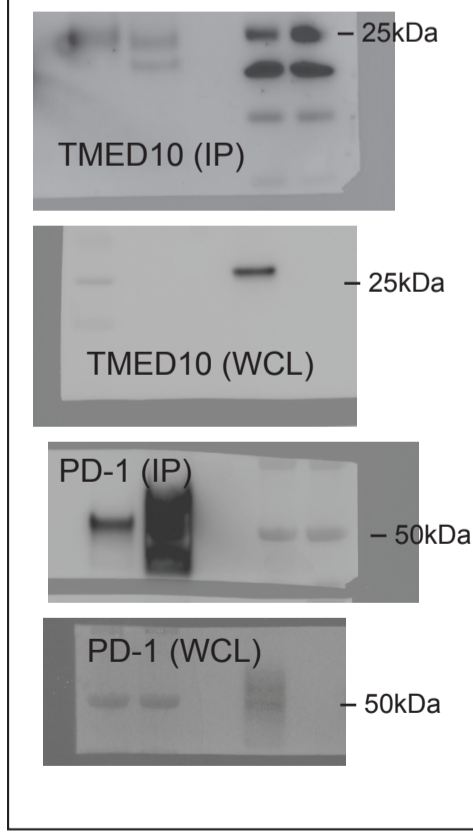

Figure S3E

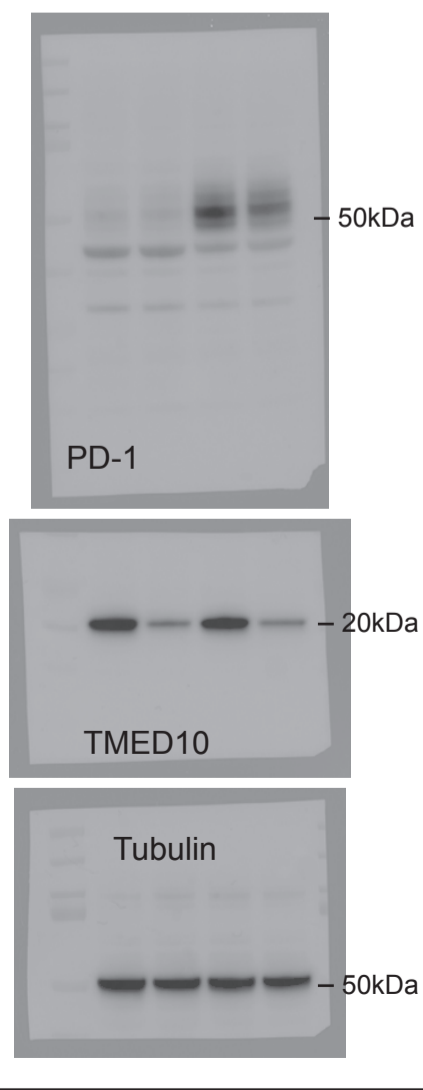

Supplement: online supplemental file 2 [file jitc-12-11-s016.pdf]
